# A WOPR transcriptional regulatory network controls the virulence of the opportunistic fungal pathogen *Nakaseomyces glabratus*

**DOI:** 10.1128/spectrum.01865-26

**Published:** 2026-09-22

**Authors:** Jian Yin, Shan Yang, Kang Xiong, Shengwei Gong, Chang Su

**Affiliations:** 1 Hubei Key Laboratory of Cell Homeostasis, College of Life Sciences, Wuhan University 98436, Wuhan, Hubei, China; University of Guelph, Guelph, Ontario, Canada

**Keywords:** *Nakaseomyces glabratus*, Wor1, WOPR, virulence

## Abstract

The WOPR family of transcription factors regulates morphological transitions and virulence in diverse fungal pathogens, yet their roles in *Nakaseomyces glabratus*—the second leading cause of candidiasis—remain uncharacterized. Through homology searches, we identified two WOPR domain-containing proteins in *N. glabratus*: Wor1 (CAGL0L02453g) and Pth2 (CAGL0C03740g), and found both are upregulated during invasive infection. Genetic deletion experiments revealed that Wor1, but not Pth2, is critical for pathogenicity. A *wor1* mutant strain showed significantly reduced fungal burden in a murine model of systemic infection and impaired adhesion to human epithelial cells. These virulence defects were not associated with changes in morphology or stress tolerance. RNA-Seq analysis of the *wor1* mutant during infection identified 400 downregulated and 175 upregulated genes, indicating that Wor1 functions primarily as a transcriptional activator. Among its targets, Wor1 directly bound the promoters of *PDR1* and *UPC2A*. Functional analysis of these downstream targets showed that Upc2A is critical for virulence and adhesion, while Pdr1 expression levels are also critical for these processes. Additionally, Wor1 regulates cell wall proteins involved in host interactions. In conclusion, our study establishes Wor1 as a pivotal regulator of a transcriptional network governing virulence in *N. glabratus*, controlling host colonization and adhesion by modulating key downstream transcription factors and cell wall components.

**IMPORTANCE:**

Fungal infections are a growing threat, especially for patients with weakened immune systems. *Nakaseomyces glabratus* is particularly dangerous because it resists many common antifungal drugs and is increasingly difficult to treat. Despite this, we know relatively little about how this pathogen causes disease. Here, we identify a critical protein, Wor1, that controls the pathogenicity of *N. glabratus*. When *WOR1* is disrupted, the fungus becomes significantly less harmful in animal models and loses much of its ability to adhere to human cells—a crucial first step for infection. Understanding how pathogens activate their disease-causing machinery is essential for finding new drug targets. Because Wor1 and the proteins it controls are specific to fungi and absent in humans, they represent promising candidates for developing therapies that attack the fungus without harming the patient.

## INTRODUCTION

The WOPR family of transcription factors constitutes a distinctive class of fungal regulators, named after its founding members: Wor1 (white-opaque regulator 1), Pac2 (pat1 compensator 2), and Ryp1 (required for yeast phase growth 1) (1–6). These proteins contain a highly conserved DNA-binding domain that is structurally divergent from other known transcription factor domains. Structural analyses at 2.6-Å resolution have revealed that the WOPR domain consists of two regions—WOPRa (approximately 80 amino acids) and WOPRb (approximately 50 amino acids)—connected by a variable-length linker (6). These regions form an interdigitated β-sheet structure that interacts with DNA in a novel manner, tilting into the major groove while establishing high-affinity interactions in the minor groove through a deeply penetrating arginine residue (7). This fungal-specific DNA-binding mechanism utilizes a conserved loop to recognize specific 6-bp DNA motifs, representing an unanticipated mode of protein-DNA interaction.

WOPR domain-containing transcription factors play crucial roles in regulating morphological transitions and virulence across diverse fungal species. In *Candida albicans*, Wor1 controls the white-opaque phenotypic switch, a process essential for mating competence and host colonization (1, 2, 8–10). Similarly, in *Histoplasma capsulatum* and *Cryptococcus neoformans*, WOPR proteins regulate morphological changes critical for pathogenesis (4, 11). In *Saccharomyces cerevisiae*, two WOPR proteins, Mit1 and Rof1, promote morphological transitions such as pseudohyphal and biofilm formation, important for adapting to environmental changes (12, 13). Notably, the WOPR protein Wor1 acts synergistically with other transcription factors to regulate gene expression in *C. albicans*. It functions at the center of a complex transcriptional regulatory network, forming positive feedback loops and interacting cooperatively with key transcription factors—such as Efg1, Wor2, Czf1, Ahr1, and Wor3—to control morphological states (6). In fact, Wor1 binds to the promoters of a majority of target genes within this regulatory circuit, underscoring its pivotal role (14). Likewise, in *S. cerevisiae*, Mit1 serves as a central regulator of filamentous growth by controlling a broad network of downstream targets, including multiple transcription factors such as Phd1, Sok2, Mga1, Flo8, Tec1, and Ste12 (12). Despite their importance in multiple fungal pathogens, WOPR domain-containing transcription factors in *Nakaseomyces glabratus* remain unidentified and uncharacterized.

*N. glabratus* has emerged as a significant opportunistic pathogen, now ranking as the second leading cause of candidiasis after *C. albicans* (15). This commensal yeast, which naturally inhabits the oral and gastrointestinal tracts of healthy individuals, can cause severe systemic infections with high mortality rates, particularly in immunocompromised patients (16). *N. glabratus* infections are challenging to treat due to the organism’s intrinsic resistance to azole antifungals and its ability to rapidly develop further resistance (17). Unlike *C. albicans*, *N. glabratus* does not typically undergo morphological transitions between yeast and hyphal forms during infection, although it can form pseudohyphae under nitrogen starvation conditions *in vitro* (18–20). The virulence of *N. glabratus* is predominantly attributed to its robust adherence capabilities, mediated by a diverse array of cell wall adhesins, particularly the epithelial adhesin (EPA) family (21–23). Furthermore, *N. glabratus* employs sophisticated strategies to sequester iron from host cells, a process essential for its pathogenicity (24, 25). Given the rising prevalence and clinical significance of *N. glabratus* infections, understanding the molecular mechanisms governing its virulence is of increasing importance.

Phylogenetic analyses indicate that *N. glabratus* is more closely related to *S. cerevisiae* than to *C. albicans* (26), raising questions about the evolutionary conservation and functional divergence of WOPR domain proteins across these species. Comparative studies have revealed that despite regulating different physiological processes, the DNA-binding specificities of WOPR proteins are largely conserved in fungi. For example, Mit1 in *S. cerevisiae* and Wor1 in *C. albicans* recognize similar DNA sequences yet control distinct sets of target genes (12). This suggests that the evolutionary diversification of WOPR-regulated processes likely results from changes in the genes under WOPR control rather than from alterations in DNA-binding specificity—a phenomenon described as the “movement of genes in and out of the WOPR regulon” (12). Whether WOPR domain proteins in *N. glabratus* regulate morphological development, host-pathogen interactions, or other virulence attributes remains unknown.

In this study, we identify WOPR domain-containing transcription factors in *N. glabratus* and elucidate their biological functions, particularly in the context of host-pathogen interactions. Based on sequence alignment, we designated CAGL0L02453g as Wor1 and CAGL0C03740g as Pth2. Deletion of *WOR1*, but not *PTH2*, resulted in reduced colonization in murine models of systemic candidiasis and diminished adhesion to human epithelial cells. We found that Wor1 binds to the promoters of Pdr1 and Upc2A, two transcription factors involved in pathogenicity, and activates their expression. Additionally, Wor1 regulates the expression of several cell wall proteins during invasive infection. Our findings establish that WOPR family proteins are critical for *N. glabratus* virulence. Given the importance of WOPR proteins in the pathogenicity of diverse fungal species, characterizing their roles in *N. glabratus* may reveal novel regulatory mechanisms controlling adhesin expression, stress responses, or other virulence-associated processes.

## RESULTS

### Identification of WOPR family transcription factors in *N. glabratus*

**Fig 1:**
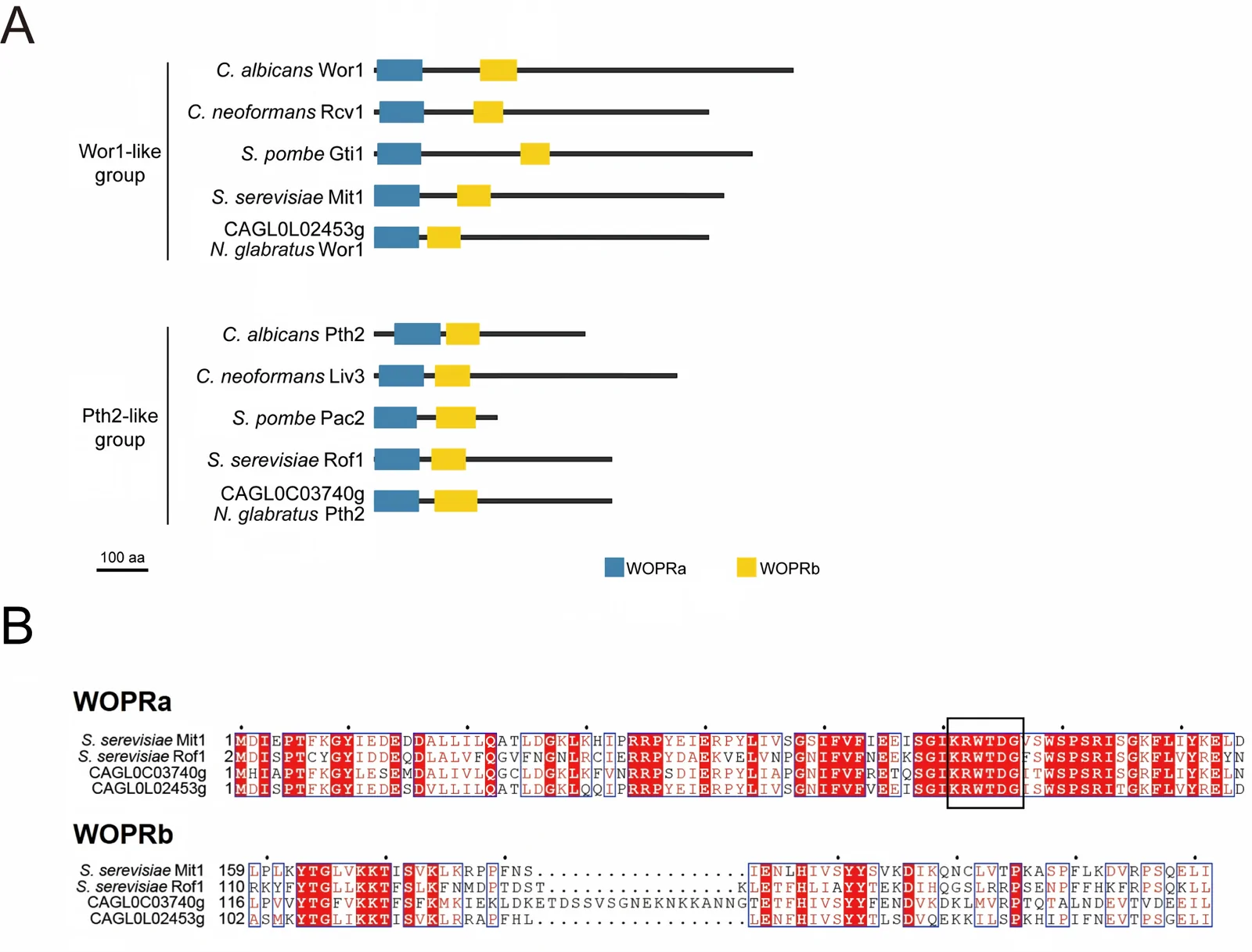
Identification and sequence analysis of WOPR family proteins in *N. glabratus*. (**A**) Domain architecture of WOPR family proteins from *N. glabratus* and representative fungal species. The schematic depicts the sequence features of WOPR homologs from *N. glabratus* and four representative fungi (*C. albicans*, *Cryptococcus neoformans*, *Schizosaccharomyces pombe*, and *S. cerevisiae*). Proteins are classified into two subgroups based on sequence homology (Wor1-like group and Pth2-like group). Blue and yellow boxes represent the highly conserved WOPRa and WOPRb domains, respectively. Scale bar, 100 aa. (**B**) Multiple sequence alignment of the N-terminal regions of WOPR homologs from *N. glabratus* and *S. cerevisiae*. Highly conserved amino acid residues are highlighted with red background and white font, while blue boxes indicate residues with similar physicochemical properties. Within the highly conserved region of the WOPRa domain, a putative PKA phosphorylation motif (KRWTDG) is identified.

To identify potential WOPR domain-containing transcription factors in the pathogenic fungus *N. glabratus*, we performed homology searches against its proteome using the resolved WOPR domain structure of *S. cerevisiae* Rof1 as a reference. This analysis identified two proteins, CAGL0L02453g and CAGL0C03740g, that contain the characteristic WOPR domain. Both proteins possess the canonical WOPRa and WOPRb conserved regions connected by a less conserved linker sequence (Fig. 1A). Further sequence analysis revealed high conservation of the WOPRa and WOPRb domains between the *S. cerevisiae* WOPR proteins (Rof1/Mit1) and the *N. glabratus* homologs (Fig. 1B). This pattern of high conservation in functional domains coupled with variability in linker regions suggests a preserved DNA-binding capability. Furthermore, a scan of the WOPRa region revealed a putative conserved phosphorylation motif for protein kinase A (PKA), KRWTDG, within this highly conserved region (Fig. 1B). In contrast to the conserved N-terminal region, the C-termini of these two proteins are not conserved, with CAGL0L02453g having a C-terminus of 463 aa and CAGL0C03740g having a much shorter one of 247 aa (Fig. 1A).

We next compared the *N. glabratus* WOPR proteins with their counterparts in *C. albicans*. A ClustalW alignment showed that the overall sequence of *C. albicans* Wor1 shares higher similarity with *N. glabratus* CAGL0L02453g (31.9%) than with CAGL0C03740g (26.4%). We therefore designated CAGL0L02453g as *N. glabratus* Wor1 (NgWor1). Phylogenetic analysis indicates that nearly all fungal species contain WOPR proteins, which can be divided into two groups, with most species having one member in each group (5, 27). *N. glabratus* Wor1 clusters in the same group as *C. albicans* Wor1, *Schizosaccharomyces pombe* Gti1, and *Cryptococcus neoformans* Rcv1 (Fig. 1A). The other group is less characterized and includes proteins like Pth2 in *C. albicans*, which is shorter than Wor1 but also contains the two conserved WOPRa and WOPRb segments. Accordingly, we designated the shorter WOPR domain-containing protein in *N. glabratus*, CAGL0C03740g, as Pth2 (NgPth2).

### Differential expression of *WOR1* and *PTH2* in *N. g* invasive infection

**Fig 2:**
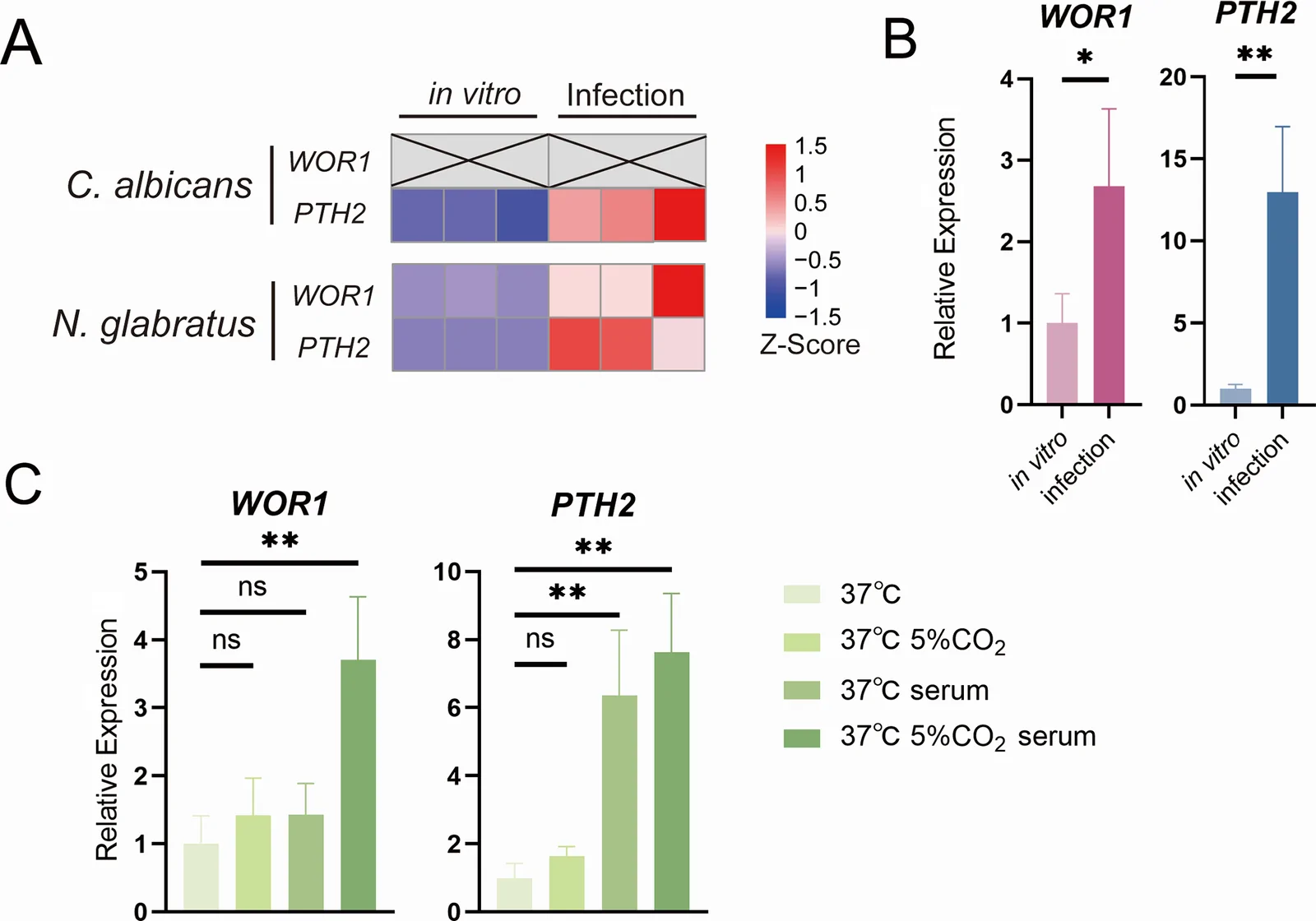
Upregulation of WOPR family genes *WOR1* and *PTH2* during invasive infection of *N. glabratus*. (**A**) Heatmap displaying transcriptional expression profiles of WOPR family genes in *C. albicans* and *N. glabratus* during murine invasive infection, with expression levels normalized as z-scores of fragments per kilobase per million mapped reads (FPKM). *In vitro*, uninfected WT *N. glabratus* cells cultured in liquid YPD medium at 30°C; infection, kidney tissue samples collected from infected mice. (**B**) qRT-PCR analysis of *WOR1* and *PTH2* expression during invasive infection of *N. glabratus*. WT cells were cultured in liquid YPD medium at 30°C as an *in vitro* control, then inoculated into 19–21 g male BALB/c mice via tail vein injection. At 24 h post-infection, total RNA was extracted from infected kidneys for *in vivo* expression analysis. *ACT1* mRNA levels were used for normalization, and data from the *in vitro* control were set to 1.00. (**C**) qRT-PCR quantification of *WOR1* and *PTH2* mRNA levels in WT *N. glabratus* cells cultured in DMEM supplemented with or without 10% serum, under air or 5% CO₂ conditions at 37°C. *ACT1* mRNA levels were used for normalization, and data from cells cultured in DMEM were set to 1.00. (**B and C**) Error bars indicate standard deviations from three independent experiments. Statistical significance was determined by unpaired *t*-test using GraphPad Prism. ns, not significant; *, *P* < 0.05; **, *P* < 0.01.

To investigate the potential role of WOPR transcription factors during host interactions, we re-examined published transcriptome data from *N. glabratus* and *C. albicans* cells isolated from infected kidneys during invasive infections (28, 29). This analysis revealed distinct expression patterns between the WOPR factors of the two species. In *C. albicans*, *WOR1* transcripts were undetectable during invasive infection (Fig. 2A), consistent with previous reports that *WOR1* expression is specific to the opaque phase, not the white cells responsible for invasive infection (2). In contrast, *WOR1* expression in *N. glabratus* was significantly upregulated at 48 h post-infection (Fig. 2A). Interestingly, the expression of *PTH2* in both *C. albicans* and *N. glabratus* was dramatically enhanced during invasive infection (Fig. 2A).

The upregulation of *WOR1* and *PTH2* in *N. glabratus* during invasive infection was validated by quantitative RT-PCR (qRT-PCR) (Fig. 2B), leading us to hypothesize that host-derived signals might activate these transcription factors. We exposed *N. glabratus* cells to various host-associated conditions and found that a combination of serum and elevated CO₂ induced significant upregulation of both *WOR1* and *PTH2* (Fig. 2C). Notably, neither serum nor high CO₂ alone was sufficient to induce *WOR1* expression, indicating that *WOR1* regulation requires the synergistic action of multiple host-associated signals. This sophisticated regulatory mechanism may allow *N. glabratus* to precisely modulate *WOR1* expression in response to the complex environmental cues encountered during infection. Collectively, these data demonstrate that WOPR transcription factors are activated during *N. glabratus* infection.

### Wor1 is critical for *N. glabratus* pathogenicity

To investigate the role of WOPR transcription factors in *N. glabratus* pathogenicity, we generated *wor1* and *pth2* single mutants, as well as a *wor1 pth2* double mutant. None of the mutants exhibited growth defects when cultured in yeast extract-peptone-dextrose (YPD) liquid medium at 30°C or 37°C (Fig. S1).

**Fig 3:**
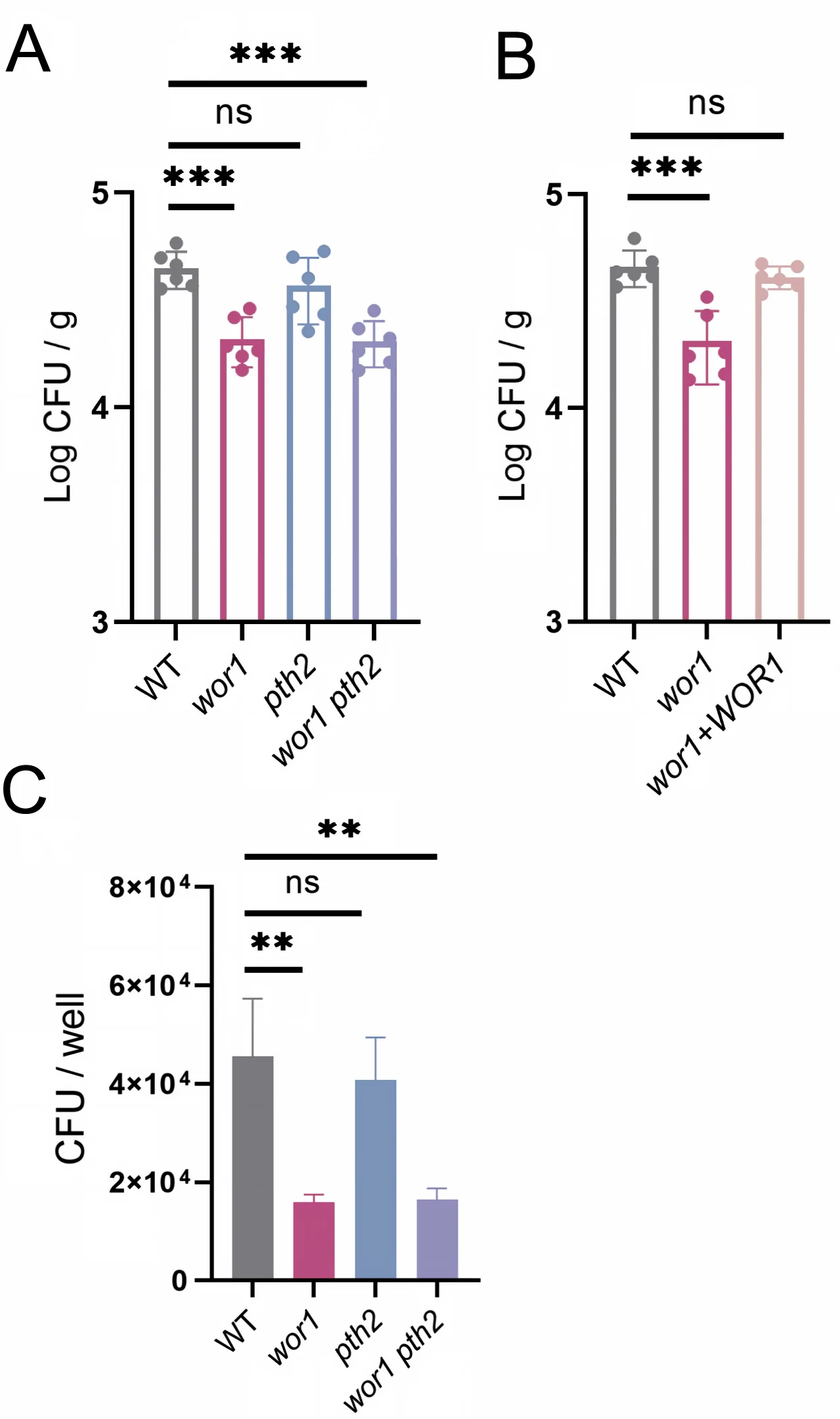
*WOR1* deletion leads to reduced virulence in invasive infection and diminished adhesion capacity to epithelial cells. (**A**) Effect of WOPR family genes on the virulence of *N. glabratus* during invasive infection. Immunosuppressed male BALB/c mice were intravenously infected via tail vein with 5 × 10^7^ CFU of WT, *wor1* single mutant, *pth2* single mutant, or *wor1 pth2* double mutant strains. Six animals per group were euthanized 4 days post-infection. Neutropenia was induced by intraperitoneal administration of cyclophosphamide (150 mg/kg of body weight per day) 3 days prior to infection and on the day of infection. Fungal burden was determined by plating kidney homogenates on agar plates containing streptomycin and ampicillin, followed by incubation at 30°C and CFU quantification. (**B**) Quantification of fungal loads in kidneys following infection with WT strain, *wor1* mutant strain, or *WOR1* complemented strain (*wor1* + pY26-WOR1) was performed as described in A. n = 6. (**C**) Adhesion assay of WOPR family gene mutants to HEK293 cells. WT and mutant *N. glabratus* strains were cocultured with HEK293 cells in DMEM supplemented with 10% fetal bovine serum for 4 h. Nonadherent fungal cells were removed by PBS washing. The number of adherent fungal cells was calculated and presented. Data are shown as means ± SD. Statistical significance was assessed by Student’s *t*-test. ns, not significant; **, *P* < 0.01; ***, *P* < 0.001.

We next assessed the survival of *N. glabratus* in the host during invasive infection. Tissue fungal burdens were measured 4 days post-infection in immunosuppressed mice following tail-vein injection. Mice infected with the *wor1* mutant exhibited significantly reduced fungal loads in the kidneys compared to those infected with the WT strain, with an approximately 2-fold reduction (Fig. 3A). This significant but modest decrease suggests that *WOR1* contributes to, but is not the sole determinant of, *N. glabratus* virulence during invasive infection. To confirm the reproducibility of this phenotype, we performed additional virulence experiments including *wor1* mutant and *WOR1* complemented strains, and found that complementation of the mutant with a wild-type *WOR1* allele rescued this defect (Fig. 3B). In contrast, deletion of *PTH2* did not significantly impact kidney colonization, as fungal burdens in mice infected with the *pth2* mutant were comparable to those of the WT (Fig. 3A). The *wor1 pth2* double mutant displayed a reduction in kidney fungal burden similar to that of the *wor1* single mutant (Fig. 3A), suggesting that the virulence defect is primarily attributable to the absence of *WOR1*. These findings indicate that while both *WOR1* and *PTH2* are upregulated during infection, only *WOR1* is necessary for *N. glabratus* survival *in vivo*. To determine whether overexpression would confer a gain-of-function phenotype, we constructed two strains (*WOR1^OE^* and *PTH2^OE^*) in which *WOR1* or *PTH2* is driven by the *TEF1* promoter. However, overexpression of *WOR1* or *PTH2* did not increase the fungal burden in murine kidneys (Fig. S2), indicating that *WOR1* is necessary but not sufficient to enhance virulence under these conditions.

Adhesion to host cells is a critical virulence attribute for *N. glabratus*, facilitated by an extensive repertoire of cell wall-associated adhesins (30, 31). To investigate potential mechanisms underlying the reduced virulence of the *wor1* mutant, we examined whether WOPR transcription factors impact the adherence of *N. glabratus* to mammalian cells. Using an established *in vitro* adhesion assay (32), we quantified the attachment of WT, *wor1*, *pth2*, and *wor1 pth2* strains to human epithelial cells. The number of adherent cells for the *wor1* single and *wor1 pth2* double mutants was approximately half that of the WT strain, indicating that deletion of *WOR1* impairs adhesion to human epithelial cells (Fig. 3C). Furthermore, the complemented strain restored adhesion to a level indistinguishable from the WT strain (Fig. S3).

**Fig 4:**
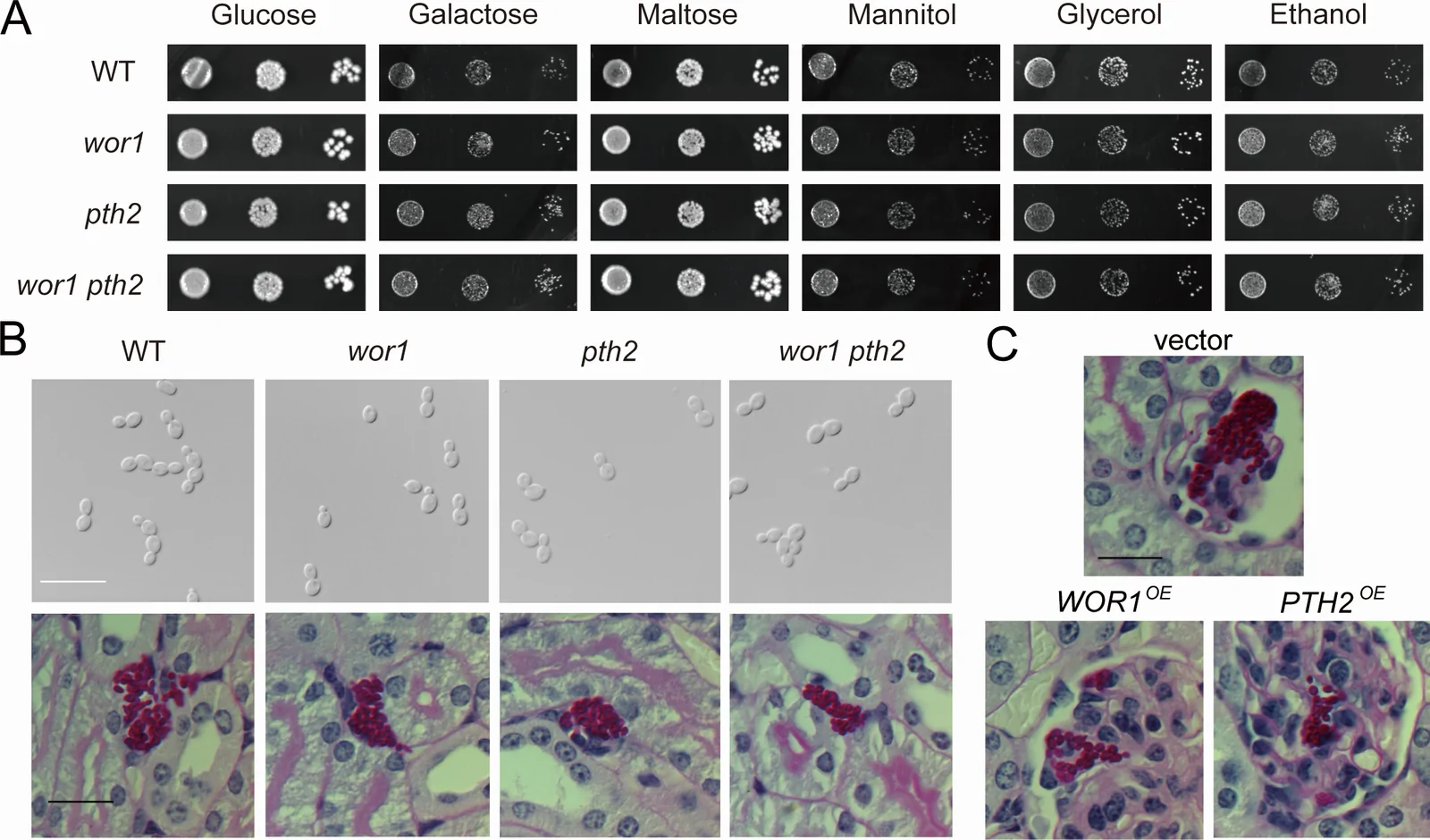
Phenotypes of WOPR family gene deletion strains on growth and morphology. (**A**) Carbon source utilization phenotypes of WOPR family gene deletion mutants. WT, single mutants (*wor1* and *pth2*), and double mutant (*wor1 pth2*) cells were serially diluted 10-fold and spotted onto YNB solid medium supplemented with 2% of the indicated carbon sources, then incubated at 30°C. (**B**) Morphological analysis of WOPR family gene deletion mutants. Upper panel: cell morphology of WT and deletion mutants (*wor1*, *pth2*, *wor1 pth2*) during log growth phase in liquid YPD medium. Lower panel: histopathological examination of *N. glabratus* morphology in infected mouse kidney tissue. PAS-stained microscopic images showing WT and deletion mutant cells within infected kidney tissue. (**C**) Morphological characterization of WOPR family gene overexpression strains during invasive infection. Scale bar, 20 μm.

Given that fungal pathogens encounter various nutrient sources within host niches, we also examined their growth on alternative carbon sources, including the fermentable sugars galactose and maltose and the non-fermentable sources glycerol and ethanol. As shown in Fig. 4A, all mutants grew comparably to the WT strain on these media. Furthermore, both single and double mutant strains maintained a yeast form similar to that of the WT strain under both *in vitro* and *in vivo* conditions (Fig. 4B). Also, histopathology revealed that the *WOR1*^*OE*^ and *PTH2*^*OE*^ strains remained predominantly in the yeast form during invasive infection (Fig. 4C). These results indicate that WOPR transcription factors do not impact the morphogenesis of *N. glabratus* in the host.

### *WOR1* is dispensable for iron acquisition and stress tolerance in *N. glabratus*

**Fig 5:**
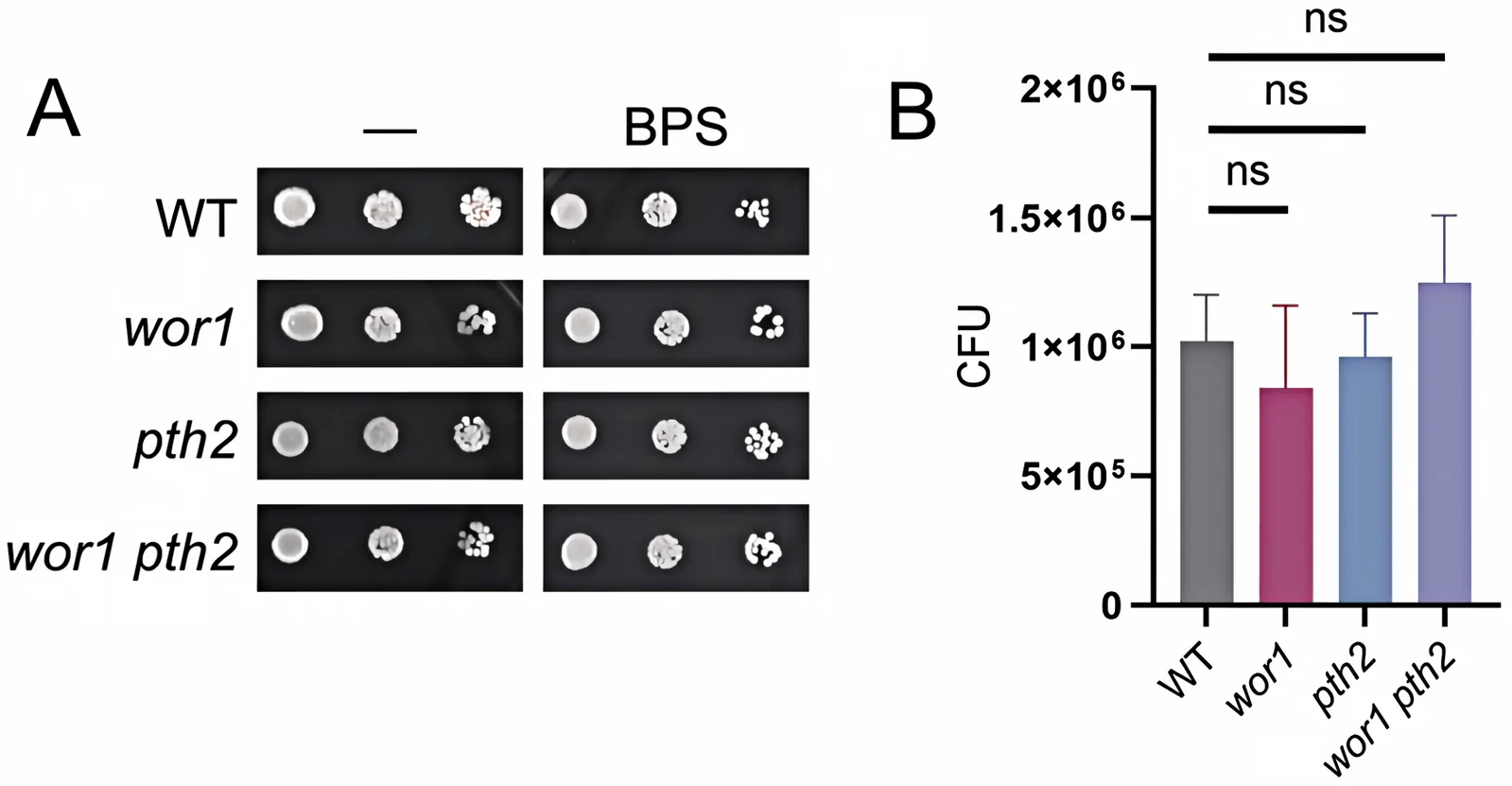
WOPR family proteins are not required for iron starvation adaptation and intracellular survival. (**A**) Role of WOPR genes in adaptation to the iron-limited condition. The WT strain and WOPR gene deletion mutants were serially diluted 10-fold and spotted onto synthetic medium (YNB with 2% glucose) without or with 25 mM BPS. Plates were incubated at 30°C for 2 days before imaging. (**B**) Impact of WOPR gene deletion on intracellular survival within macrophages. WT, *wor1*, *pth2*, and *wor1 pth2* strains were cocultured with RAW264.7 macrophages at a MOI of 3:1 for 2 h, followed by removal of extracellular fungi by washing. After an additional 6-h incubation, host cells were lysed and intracellular fungal viability was quantified by CFU assays. Experiments were performed in triplicate biological replicates. Data are shown as means ± SD. ns, no significance.

Fungal pathogens require efficient mechanisms to acquire iron from host environments (33, 34). To investigate whether Wor1 is important for iron metabolism, we compared the growth of WT and mutant strains under iron-limited conditions. When cultured on media containing the iron-specific chelator bathophenanthroline disulfonate (BPS), the WT, *wor1*, and *pth2* strains exhibited comparable growth (Fig. 5A), suggesting that *WOR1* and *PTH2* are not essential for iron acquisition. We next evaluated the growth of these mutants under various stress conditions. The single and double mutants demonstrated normal growth patterns when exposed to osmotic stress (2.5 M KCl), oxidative stress (10 mM H₂O₂), and cell wall stress (4 mg/mL Congo red) compared to the wild-type strain (Fig. S4A and B). No significant growth differences were observed under heat stress at 42°C (Fig. S4C). These findings suggest that *WOR1* and *PTH2* are not involved in the responses to the stresses we tested. Furthermore, Wor1 and Pth2 did not impact *N. glabratus* survival in macrophages (Fig. 5B).

### RNA-Seq analysis reveals genes regulated by Wor1 in *N. glabratus*

**Fig 6:**
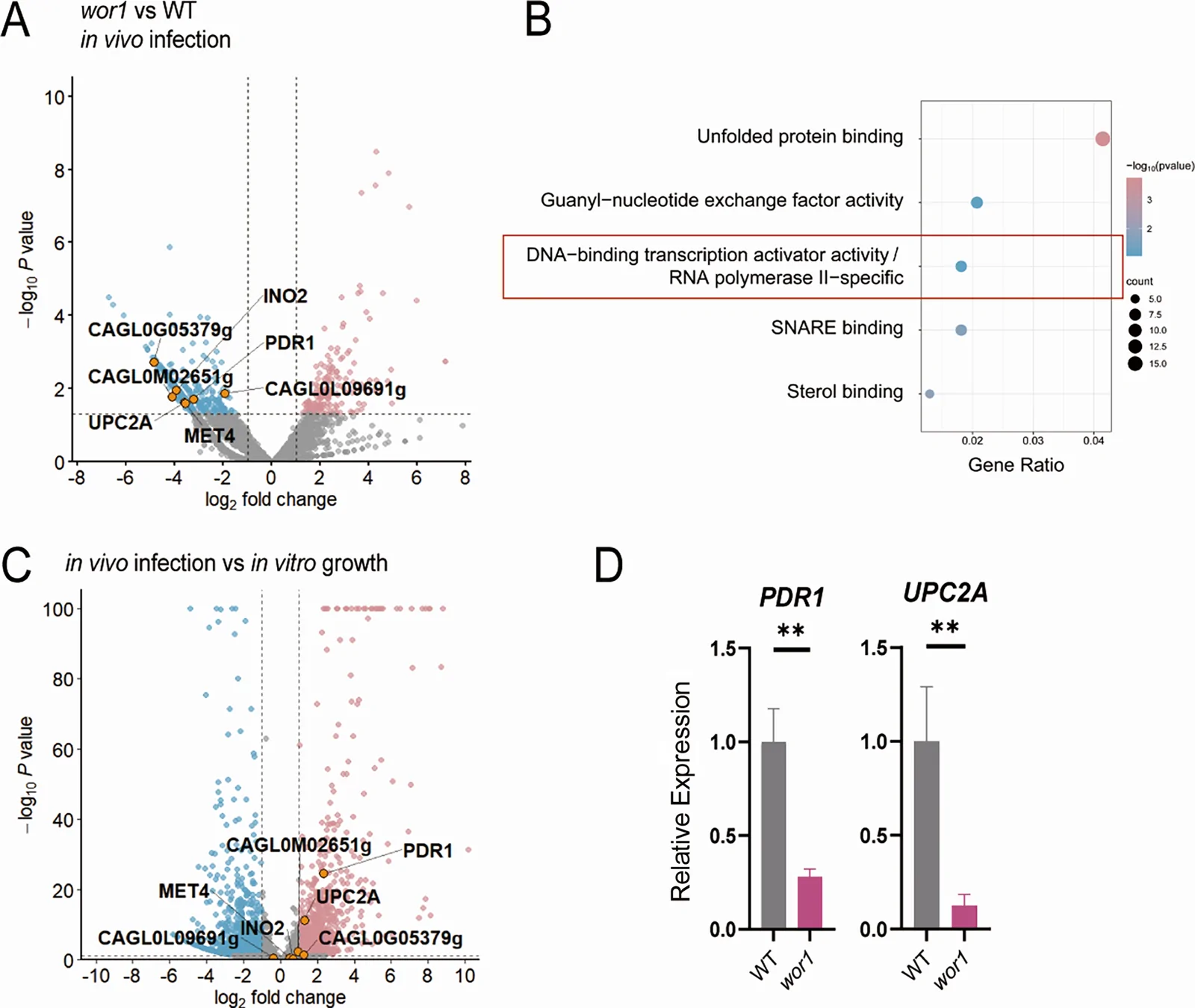
Differential gene expression analysis in the *wor1* mutant during invasive infection. (**A**) Volcano plot of differentially expressed genes between WT strain and *wor1* mutant under *in vivo* infection conditions. Genes with |log_2_ fold change| ≥ 1 and *P* < 0.05 were considered significant. Red dots indicate significantly upregulated genes, blue dots represent significantly downregulated genes, and gray dots denote genes without significant expression changes. (**B**) GO enrichment analysis of Wor1-activated genes during invasive infection. The x-axis shows the Gene Ratio (proportion of genes enriched), bubble size corresponds to the number of genes assigned to each term, and bubble color reflects the statistical significance of enrichment. (**C**) Volcano plot comparing gene expression profiles of *N. glabratus* in *in vivo* infection versus *in vitro* culture in YPD medium at 30°C. The x-axis depicts log_2_ fold change, and the y-axis indicates significance level. Orange points represent candidate transcription factors identified based on GO enrichment for DNA-binding transcription activator activity in B. The same thresholds and color coding were used as in A. (**D**) qRT-PCR validation of the expression levels of transcription factors Pdr1 and Upc2A during infection. Total RNA extracted from murine kidneys at 24 h post-infection was used to quantify relative expression levels of *PDR1* and *UPC2A* in WT strain and *wor1* mutant. Expression was normalized to *ACT1,* and data from the WT control were set to 1.00. Data represent means ± SD from three replicates. Statistical significance was assessed by Student’s *t*-test. **, *P* < 0.01.

To further define the function of Wor1, we compared the transcriptional profiles of the *wor1* mutant and the WT strain during invasive infection in a murine model using RNA-Seq. Quality metrics for the transcriptome sequencing data are shown in Fig. S5. Using a 2-fold expression change cutoff, we identified genes that were differentially expressed in the *wor1* mutant (Table S1). A volcano plot revealed that deletion of *WOR1* resulted in the downregulation of 400 genes and upregulation of 175 genes relative to the WT strain during invasive infection (Fig. 6A), indicating that Wor1 primarily functions as a transcriptional activator in *N. glabratus*. Similar to its homolog in *C. albicans*, the Wor1-activated genes in *N. glabratus* were significantly enriched for the gene set associated with “DNA-binding transcription activator activity” (Fig. 6B). Among the six transcription factors activated by Wor1, Pdr1 and Upc2A were also significantly upregulated during invasive infection as seen in previously published transcriptome data (28) (Fig. 6C). The activation of Pdr1 and Upc2A by Wor1 during *in vivo* infection was validated by qRT-PCR (Fig. 6D).

### Wor1 activates Pdr1 and Upc2A to promote *N. glabratus* virulence

**Fig 7:**
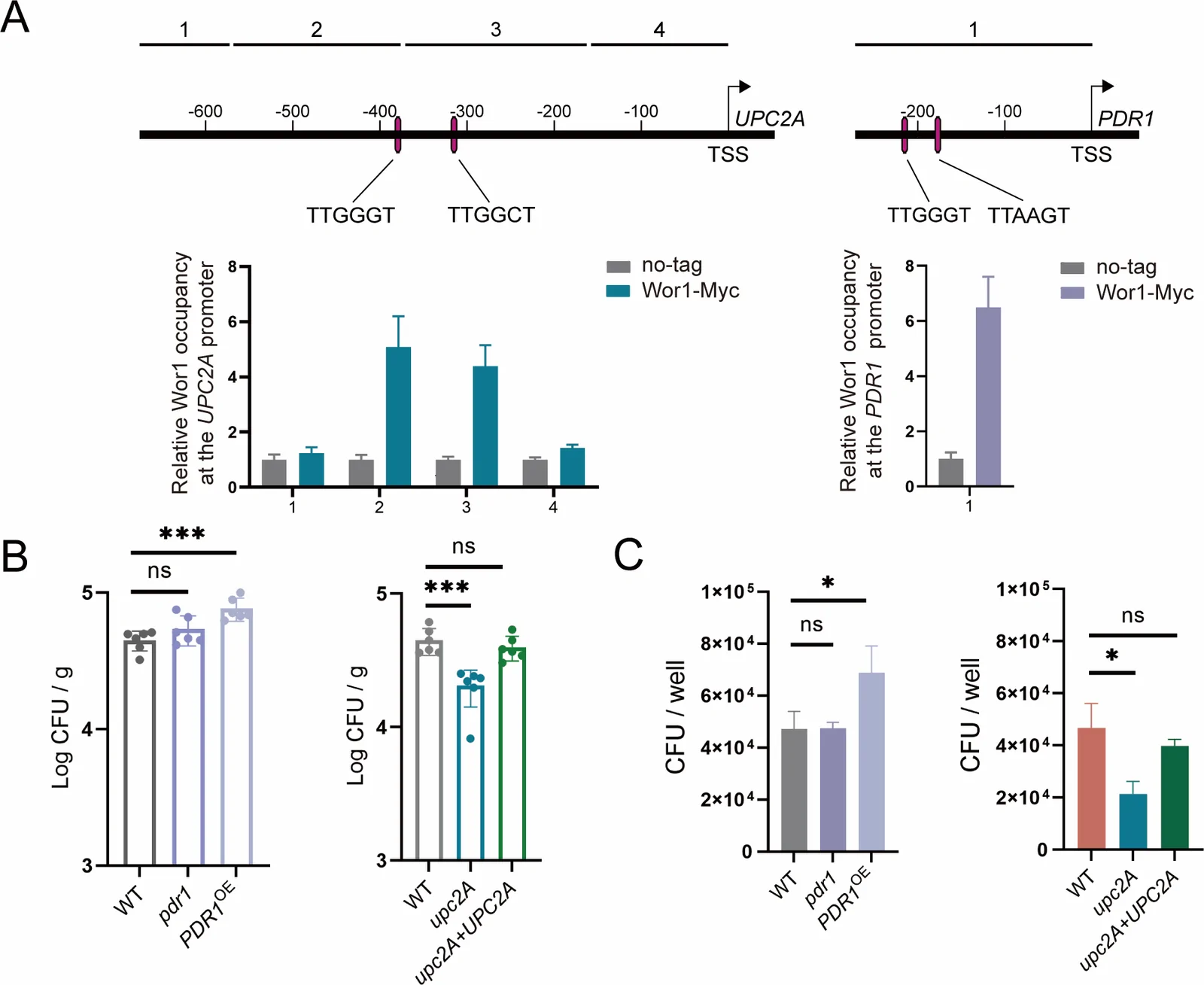
Wor1 binds the promoters of Pdr1 and Upc2A to promote *N. glabratus* virulence. (**A**) Enrichment of Wor1 at the *PDR1* and *UPC2A* promoter regions. Wild-type *N. glabratus* strains expressing Myc-tagged Wor1 or untagged Wor1 under the control of the *TEF1* promoter were cultured to log phase and processed for ChIP. DNA recovered from ChIP was quantified by qPCR using primers targeting promoter regions of *PDR1* and *UPC2A*, with the *ACT1* coding region serving as a negative binding control. Enrichment was calculated as the ratio of bound to input DNA for *PDR1* or *UPC2A* promoters normalized against the *ACT1* control, and data from untagged strains were set to 1.00. The results represent the mean of three independent experiments, with error bars denoting standard deviation. The panel above presents an analysis of the sequences between the transcription start sites of *UPC2A* and *PDR1* and their upstream neighboring genes, identifying potential Wor1-binding motifs. (**B**) Influence of *PDR1* and *UPC2A* on *N. glabratus* virulence in a murine invasive infection model. Immunosuppressed BALB/c mice were intravenously inoculated via tail vein with 5 × 10^7^ CFU of WT, *PDR1* deletion, *PDR1* overexpression, *UPC2A* deletion, or *UPC2A*-complemented strains. The experiment was performed as described in Fig. 3A. Data represent means ± SD from six mice per group. Statistical significance was assessed by Student’s *t*-test. ns, no significance; ***, *P* < 0.001. (**C**). Adhesion capacity of *N. glabratus* strains to HEK293 cells. Fungal cells from the strains described in B were co-incubated with HEK293 cells in DMEM containing 10% fetal bovine serum for 4 h. Following incubation, nonadherent fungi were removed by PBS washes. Adherent fungal cells were quantified, and data are presented as means ± SD from three independent experiments. Statistical significance was assessed by Student’s *t*-test. ns, not significant; *, *P* < 0.05.

To determine whether Wor1 directly regulates the expression of Pdr1 and Upc2A, we performed chromatin immunoprecipitation (ChIP) assays. ChIP followed by quantitative PCR (ChIP-qPCR) revealed that Wor1-Myc is associated with the promoter regions of both *PDR1* and *UPC2A* (Fig. 7A). These Wor1-bound regions contain sequences matching the reported Wor1 binding motif, T[TA][AG][AG][CG]T (7), suggesting direct transcriptional regulation.

Previous studies have demonstrated that gain-of-function mutations in *PDR1* enhance adhesion to epithelial cells (35). However, the role of Upc2A in *N. glabratus*-host interactions remains less understood. To elucidate their functional contributions, we generated *PDR1* and *UPC2A* knockout strains. Deletion of either gene had no effect on growth in YPD liquid medium (Fig. S6). The *pdr1* mutant exhibited virulence and epithelial cell adhesion comparable to the WT strain (Fig. 7B and C). In contrast, deletion of *UPC2A* resulted in a significant reduction in kidney fungal burden during invasive infection (Fig. 7B) and diminished adhesion to host epithelial cells (Fig. 7C). Interestingly, although loss of *PDR1* did not impair virulence, overexpression of *PDR1* markedly increased both kidney fungal burden and adhesion capacity (Fig. 7B and C), highlighting the importance of *PDR1* expression levels for virulence. These findings suggest that Wor1 promotes *N. glabratus* virulence, at least in part, through its regulation of Pdr1 and Upc2A.

Given that *WOR1* regulates the expression of *PDR1* and *UPC2A*, both of which are well-established regulators of azole antifungal susceptibility, we tested fluconazole susceptibility in the *wor1* single mutant, *pth2* single mutant, and *wor1 pth2* double mutant. The results demonstrated that the susceptibility of all deletion mutants to fluconazole was comparable to that of the WT strain (Fig. S7A). To gain further insight into the functional scope of Wor1-regulated genes, we performed cellular component enrichment analysis. This revealed a significant enrichment of Wor1 target genes associated with the “cell periphery” (Fig. S7B), consistent with Wor1’s role in modulating adhesion. Notably, Wor1 controls the expression of members of the YPS gene cluster, which is crucial for cell wall remodeling, including *YPS3, YPS5, and YPS11*. Altered expression of cell wall structural proteins Tir1, Tir4-5, and Cwp1.2 was also detected in the *wor1* mutant during infection. qRT-PCR confirmed the Wor1-dependent regulation of these genes (Fig. S7C). Given that previous studies suggest alterations in cell wall protein expression facilitate host cell adhesion and organ persistence by regulating Epa1 surface exposure in *N. glabratus* (23, 36), we further investigated whether Wor1 modulates the cell surface localization of the adhesin Epa1. Immunofluorescence analysis revealed a significantly reduced surface exposure of Epa1 in the *wor1* mutant compared to the WT strain (Fig. S7D), suggesting that the attenuated virulence observed in the *wor1* mutant may be attributable, at least in part, to impaired cell surface presentation of Epa1. Collectively, these findings indicate that Wor1 contributes to virulence by modulating the expression of cell wall-associated proteins, thereby influencing adhesin accessibility at the cell periphery.

## DISCUSSION

WOPR transcription factors play a crucial role in fungal morphogenesis. In the major human pathogen *C. albicans*, the WOPR transcription factor Wor1 is a master regulator of the white-opaque phenotypic switch (2). However, given that *C. albicans* predominantly exists in the white state during invasive infection, it has been suggested that Wor1 is unlikely to participate in pathogenic mechanisms underlying host invasion. Our sequence analysis confirms that *N. glabratus* harbors two WOPR domain-containing transcription factors, Wor1 and Pth2, consistent with the genomic architecture observed in most fungal species. Our study reveals that both *WOR1* and *PTH2* exhibit significantly elevated expression during invasive infections. This is noteworthy because WOPR transcription factors are commonly regulated at the transcriptional level. For instance, *WOR1* in *C. albicans* and *RYP1* in *H. capsulatum* exhibit stringent transcriptional control, with minimal expression during the white and filamentous phases, respectively, but high induction following a shift to the opaque or yeast phase (2, 4). Our study revealed elevated mRNA levels of Wor1 and Pth2 during invasive infections in *N. glabratus*. Previous studies have demonstrated that the intergenic region preceding *WOR1* in *C. albicans* is unusually large and regulated by multiple transcription factors (14). Similarly, *N. glabratus WOR1* is preceded by an exceptionally large 11.3-kb intergenic region, suggesting that complex regulatory mechanisms may modulate its expression. Notably, we observed that overexpression of *WOR1* did not enhance virulence, implying that Wor1 function may require cofactors or post-translational modifications for full activity. Indeed, a conserved potential phosphorylation site for protein kinase A (37), a characteristic feature of WOPR proteins, is present in both Wor1 and Pth2 of *N. glabratus*.

In *Candida tropicalis*, a phylogenetically conserved Wor1 ortholog similarly modulates white-opaque switching (38). Recently, it was reported that Wor1 is a hallmark transcriptional regulator of white-brown switching in *Candida auris* (39). In other fungal species, WOPR transcription factors do not govern white-opaque switching but still serve as key regulators of cellular morphology. For instance, in *S. cerevisiae* and *H. capsulatum*, these factors orchestrate the transition between budding yeast and filamentous forms (4, 12). Given that the white-opaque switch appears to be a relatively recent evolutionary adaptation in the ascomycete lineage, it has been hypothesized that the primordial role of WOPR transcription factors was to govern broader morphological shifts, such as those between yeast and filamentous states. Morphological transitions are vital for virulence in many pathogenic fungi. However, although *N. glabratus* can adopt a pseudohyphal form under nitrogen-limiting conditions *in vitro*, this filamentation has not been documented *in vivo* (18–20). Thus, *N. glabratus* does not depend on reversible switches between yeast and hyphal/pseudohyphal morphologies for its pathogenicity, and morphological switching is not a primary regulatory target of WOPR transcription factors.

In *N. glabratus*, deleting *WOR1*, but not *PTH2*, substantially impaired fungal survival during invasive infection, supporting a critical role for Wor1 in virulence. This suggests that Wor1 and Pth2 have distinct regulatory functions. Interestingly, Mit1 and Rof1, the two WOPR transcription factors in *S. cerevisiae*, regulate differential physiological processes: Mit1 functions as a transcriptional activator driving filamentous growth, while Rof1 acts as a regulator of fluffy colony morphology (12, 13). In *C. albicans*, Wor1 is essential for white-opaque switching and gut colonization, whereas the role of Pth2, the other WOPR transcription factor, remains poorly understood (3). It seems that the Pth2-related family is considerably less characterized than the Wor1-like proteins across various fungal species (5).

Wor1-like proteins are implicated in virulence in numerous phytopathogenic fungi, including *Fusarium oxysporum*, *Fusarium graminearum*, *Beauveria bassiana*, and *Cryphonectria parasitica* (27, 40–42). These transcriptional regulators modulate pathogenicity through diverse mechanisms, such as controlling fungal development, orchestrating asexual and sexual sporulation processes, regulating the production of effector molecules, and coordinating responses to environmental stressors. Remarkably, despite their evolutionarily conserved function in fungal virulence against plant hosts, these Wor1-like proteins exhibit substantial divergence regarding their downstream regulatory targets across different fungal species. Even in closely related fungal species such as *F. oxysporum* and *F. graminearum*, very little overlap was found among orthologous genes regulated by their respective Wor1-like proteins (40). Similarly, within the Saccharomycotina subphylum, although studies have established that *C. albicans* Wor1 and its *S. cerevisiae* ortholog Mit1 recognize identical DNA motifs, the transcriptional networks they govern share limited overlap. Structural biology investigations and DNA-binding assays have demonstrated that the WOPR domain, which functions as the DNA-binding module within these proteins, exhibits remarkable conservation in its target sequence recognition (7). This suggests that the substantial reprogramming of transcriptional networks observed across species occurs primarily through evolutionary divergence in the downstream gene targets rather than alterations in DNA-binding specificity. In our current investigation, we demonstrate that *N. glabratus* Wor1 does not regulate cellular growth or stress responses. Functional assays revealed that, although *WOR1* deletion did not affect survival within macrophages, it significantly impaired adhesion to epithelial cells. Given that epithelial adhesion is a critical early step in establishing an invasive infection, the attenuated virulence of the *wor1* mutant may be attributable, at least in part, to this impaired colonization capacity.

*C. albicans* Wor1 orchestrates phenotypic switching through a network of interlocking positive feedback loops involving multiple transcriptional regulators, including Czf1, Efg1, and Wor2 (14). In this study, transcriptomic analysis revealed that Wor1 regulates the expression of multiple transcriptional regulators during invasive infection of *N. glabratus*. Notably, the transcription factors Pdr1 and Upc2A were significantly upregulated during invasive infection in a Wor1-dependent manner. Pdr1 is a well-characterized regulator of ABC-transporter genes (*CDR1*, *CDR2*, and *SNQ2*) that mediate azole resistance (43–45). Previous studies have demonstrated that gain-of-function mutations in *PDR1* enhance both virulence and epithelial cell adhesion (35, 46). Our functional analysis extends these findings by demonstrating that, while *PDR1* deletion does not impair virulence or epithelial adhesion, elevated *PDR1* expression significantly promotes both phenotypes. Upc2A, a regulator of ergosterol biosynthesis and sterol biosynthesis that confers azole resistance, has not been previously linked to virulence in *N. glabratus* (47). Our findings demonstrate a critical role of Upc2A in virulence and host cell adhesion, suggesting that azole resistance mechanisms in *N. glabratus* may be coupled with pathogenicity. We further demonstrated that *N. glabratus* Wor1 binds to the promoter regions of *PDR1* and *UPC2A*. Intriguingly, Upc2A has been reported to bind the *PDR1* promoter (48), raising the possibility that Wor1 and its downstream targets, Pdr1 and Upc2A, form an interconnected positive feedback network. This regulatory architecture would parallel the well-characterized white-opaque switching circuit in *C. albicans*, where Wor1 occupies a central position within a network of positive feedback loops. Comparison of the Wor1 regulon with published Upc2A and Pdr1 target gene sets revealed that their shared targets are not enriched in the antifungal drug resistance pathways classically associated with these two transcription factors (49, 50), consistent with the observation that deletion of *WOR1* does not alter *N. glabratus* susceptibility to fluconazole. Instead, the overlap between Wor1 and Upc2A is concentrated in cell wall-related genes, suggesting that Wor1 promotes virulence through modulation of cell wall organization via a subset of Upc2A targets. It should be noted that the RNA-Seq data in this study were derived from kidney tissue samples obtained during invasive infection, in which host-derived reads constitute the majority of the sequenced library. As a result, the proportion of reads mapping to *N. glabratus* was limited. Consequently, the transcriptomic comparisons presented here are primarily informative for moderately to highly expressed *N. glabratus* genes. The precise contributions of Wor1, Pdr1, and Upc2A to this regulatory process warrant further investigation. Our study provides new insight into how conserved transcription factor families can be co-opted to regulate species-specific virulence programs.

## MATERIALS AND METHODS

### Media and growth conditions

*N. glabratus* strains were routinely grown at 30°C in YPD medium (2% Bacto peptone, 2% glucose, 1% yeast extract). Transformants were selected on YPD plates supplemented with 80 μg/mL nourseothricin or synthetic medium (0.17% Difco yeast nitrogen base w/o ammonium sulfate, 0.5% ammonium sulfate, and auxotrophic supplements) with 2% glucose.

The ability of *N. glabratus* cells to grow was tested by spotting dilutions of cells onto YNB (0.17% Difco yeast nitrogen base w/o ammonium sulfate, 0.5% ammonium sulfate) solid medium with 2% of different sugars followed by incubation at 30°C. To determine the stress response of *N. glabratus*, freshly cultured cells were serially diluted 10-fold, spotted onto YPD plates supplemented with 4 mg/mL Congo red, 2.5 M KCl, or 10 mM H_2_O_2_, and incubated at 30°C. To evaluate the growth under the iron-depleted condition, *N. glabratus* cells were subjected to 10-fold serial dilutions, and then spotted onto YNB medium with 2% glucose in the presence of 25 mM BPS. To analyze the heat stress response of *N. glabratus*, fresh cells were 10-fold serially diluted and spotted on YPD plates, followed by incubation at 30°C (permissive) and 42°C (stress condition), respectively.

To generate growth curves of *N. glabratus*, overnight cultures grown in YPD medium were diluted to an initial optical density at 600 nm of 0.1 in 25 mL of fresh sterile YPD medium within Erlenmeyer flasks. The cultures were then incubated at 30°C with continuous shaking. Optical density measurements at 600 nm were taken hourly to monitor cell growth.

### Plasmid and strain construction

CBS138 genomic DNA was used as the template for all PCR amplifications of *N. glabratus* genes. *N. glabratus* strains used in this study are listed in Table S2. The primers used for PCR amplifications are listed in Table S3.

Deletion of *WOR1* (*CAGL0L02453g*), *PTH2* (*CAGL0C03740g*), *PDR1*, and *UPC2A* was performed using CRISPR-Cas9 strategy as follows. The single-guide RNA (sgRNA) was annealed to insert into the pV1382 vector (51). The resulting plasmid was transformed into *N. glabratus* cells with the repair template. The mutants were verified by sequencing. *PDR1* overexpression strain was constructed using a CRISPR-Cas9-based strategy. Briefly, the single-guide RNA targeting the upstream region of the *PDR1* locus was generated by annealing primers 17 and 18 and cloned into the pV1382 vector. The resulting plasmid was co-transformed into *N. glabratus* cells together with a repair template containing the *TEF1* promoter, which was amplified from plasmid pY26TEF-GPD using primers 19 and 20. Transformants were screened by PCR, and successful overexpression of *PDR1* was confirmed by qRT-PCR.

The coding sequences for *WOR1*, *PTH2*, and *UPC2A* were amplified from *N. glabratus* genomic DNA. The PCR product was then inserted into the NotI-SacII site of pY26TEF-GPD, generating the plasmid to express *WOR1*, *PTH2*, or *UPC2A* under the control of TEF1p in *N. glabratus*. The coding sequence of *WOR1* was amplified from *N. glabratus* genomic DNA using primers 25 and 26. The resulting PCR product was cloned into the pYC121 vector, which had been linearized with XbaI and BamHI, to generate the Myc-tagged fusion expression plasmid pYC121-WOR1.

### RNA extraction and quantitative PCR expression analysis

To determine the expression of *N. glabratus* genes during invasive infection, 19–21 g male BALB/c mice were inoculated with 6 × 10^8^ live *N. glabratus* cells by tail vein injection, and infected kidneys were taken at 24 h post-infection. Kidneys were immediately placed in a prechilled mortar and ground into a fine powder under liquid nitrogen. The homogenized tissue was then transferred to a microcentrifuge tube, and 1 mL of lysis buffer (0.1% Triton X-100) was added. The mixture was incubated on ice for 1 min to selectively lyse host cells, followed by centrifugation at 3,000 rpm for 5 min at 4°C. The supernatant was discarded, and the pellet was resuspended in fresh lysis buffer. This lysis and centrifugation procedure was repeated once to maximize the removal of host cellular material and enrich for fungal cells. Total RNA was then extracted from the enriched fungal pellet using the RNAprep Pure Tissue Kit (Tiangen) according to the manufacturer’s instructions. The total RNA from *N. glabratus* cells cultured under *in vitro* conditions was extracted directly using the RNAprep Pure Tissue Kit (Tiangen). cDNA was synthesized using the Maxima H Minus cDNA Synthesis Master Mix with dsDNase (Thermo). qRT-PCR analysis was performed using iQ SYBR Green Supermix (Bio-Rad) with gene-specific primers listed in Table S3. Gene expression levels were normalized to *ACT1* mRNA signals.

### *In vivo* RNA sequencing and analysis

DNA-depleted RNA samples were then depleted of ribosomal RNA using the Ribo-Zero rRNA Removal Kit (Epicenter) according to the manufacturer’s protocol. Sequencing was performed using the Illumina NovaSeq 6000 platform. Clean reads were selected from raw reads by removing reads with adapters and low quality. Q30 and GC content of clean data were calculated. The sequencing reads were then aligned to the *N. glabratus* reference genome (http://www.candidagenome.org/) using HISAT2 with default parameters, and the aligned reads were assembled and quantified. Read counts were normalized using the variance-stabilizing transformation implemented in DESeq2, which accounts for differences in library size and is robust to samples with heterogeneous sequencing depth. DESeq2’s negative binomial model internally corrects for sequencing depth via size factor estimation, allowing statistically valid comparisons across samples with unequal read numbers. Differentially expressed genes were defined by a fold changeof ≥2 and a false discovery rate of <0.05 in DESeq2. Gene Ontology (GO) enrichment analysis was performed using DAVID (https://davidbioinformatics.nih.gov/DAVID) and Candida Genome Database (https://www.candidagenome.org/go-term-finder). RNA-Seq data that support the findings of this study have been deposited in the Gene Expression Omnibus under the accession code GSE342733.

### Infection of macrophages

RAW264.7 cells were challenged with *N. glabratus* at a MOI of 1:3 (macrophage:yeast). Nonphagocytosed yeast cells were removed by washing with PBS after co-incubation for 2 h. To determine the growth of intracellular yeast cells, RAW264.7 cells were lysed with 0.1% Triton after incubation for an additional 6 h. After resuspension, serial dilution, and plating onto YPD plates, the phagocytized yeast cells were counted. The results are represented as means ± SD from three independent experiments.

### Adhesion of *N. glabratus* to human epithelial cells

HEK293 cells were cultured in DMEM supplemented with 10% fetal bovine serum (FBS) at 37°C in 5% CO_2_. For the adhesion assay, *N. glabratus* cells suspended in DMEM containing 10% FBS were added to the 24-well plate where HEK293 cell monolayers had been prepared, at a MOI of 1:4 (HEK293:yeast). After incubation at 37°C for 4 h, nonadherent *N. glabratus* cells were carefully removed by washing with PBS. Subsequently, host cells were lysed by treatment with 0.1% Triton X-100 to release *N. glabratus* cells. The lysate was then washed with PBS to remove residual detergent and diluted. Finally, the diluted samples were plated on agar plates and incubated to quantify the number of adherent *N. glabratus* cells.

### Quantification of fungal burden in a murine model of systemic *N. glabratus* infection

BALB/c mice were purchased from Hunan SJA Laboratory Animal Company. Mice were housed in a temperature-constant animal room (22°C) with a reversed dark/light cycle (7:00 a.m. on and 7:00 p.m. off) and 40%–70% humidity.

Neutropenia was induced in 19–21 g male BALB/c mice by administering cyclophosphamide intraperitoneally (150 mg/kg of body weight) 3 days before challenge with *N. glabratus* infection and on the day of infection. For the infection, *N. glabratus* cells were delivered into the immunosuppressed mice through a tail vein injection at a dosage of 5 × 10^7^ cells. On day four post-infection, the infected kidneys from these mice were harvested, homogenized, and cultured on agar plates supplemented with streptomycin (100 μg/mL) and ampicillin (50 μg/mL). Fungal colony-forming units (CFUs) were counted after incubation at 30°C.

### Histology

For histological analysis, male BALB/c mice weighing 19–21 g were intravenously infected via the tail vein with 6 × 10^8^ *N. glabratus* cells. After 24 h, three kidneys per strain were harvested, fixed in 4% paraformaldehyde for 12 h, and subsequently embedded in paraffin. Thin sections were then prepared and stained using the periodic acid–Schiff (PAS) method for microscopic examination.

### Chromatin immunoprecipitation

*N. glabratus* cells were grown to the log phase and collected for chromatin immunoprecipitation (ChIP). DNA was sheared by sonication six times for 10 s at high power on a Bioruptor (Diagenode) with 20-s intervals. For immunoprecipitation (IP), 5 μL of anti-Myc antibody was utilized for ~2 mg of chromatin proteins, and the volume of immunoprecipitation was 300 μL. The ChIP DNA was quantified by qPCR with primers specific to the *PDR1* or *UPC2A* promoters. The coding region of *ACT1* served as a negative control. The enrichment is presented as a ratio of *PDR1* or *UPC2A* promoter IP (bound/input) versus *ACT1* IP (bound/input) of the tagged strain (Wor1-Myc), and further normalized to the control strain.

### FACS assay

Surface exposure of Epa1 was quantified by flow cytometry following the previously established protocol (23). Briefly, HA-tagged Epa1-expressing WT and *wor1* mutant cells were cultured in DMEM supplemented with 10% serum at 37°C under 5% CO_2_. For immunolabeling, cells were harvested and incubated with a mouse anti-HA primary antibody (MBL) for 30 min at 4°C with gentle agitation. Subsequently, samples were labeled with a FITC-conjugated goat anti-mouse secondary antibody (ABclonal). A negative control lacking the primary antibody was included to account for nonspecific binding. After three washes in PBS, the *N. glabratus* cells were resuspended in 250 µL of PBS and analyzed on a CytoFLEX flow cytometer (Beckman).

### Quantification and statistical analysis

All experiments were performed with at least three biological repeats except as indicated in the figure legends, and no statistical method was used to predetermine sample sizes. Analyses were conducted using GraphPad Prism 9.0 software. Results are expressed as the mean ± SD as indicated and analyzed using Student’s *t*-test or log-rank test. *P* values of less than 0.05 were considered statistically significant. ns, no significance; *, *P* < 0.05; **, *P* < 0.01; ***, *P* < 0.001; ****, *P* < 0.0001.

## Data Availability

RNA-Seq data that support the findings of this study have been deposited in the Gene Expression Omnibus under the accession code GSE342733. All data reported in this paper will be shared by the corresponding author upon request.

## SUPPLEMENTAL MATERIAL

Supplemental material spectrum.01865-26-s0001.pdf Fig. S1 to S7; Tables S2 and S3.

Table S1 spectrum.01865-26-s0002.xls Differentially expressed genes in *wor1* mutant during invasive infection compared to WT.

ASM does not own the copyrights to Supplemental Material that may be linked to, or accessed through, an article. The authors have granted ASM a non-exclusive, world-wide license to publish the Supplemental Material files. Please contact the corresponding author directly for reuse.

## References

[1] Zordan RE, Galgoczy DJ, Johnson AD. 2006. Epigenetic properties of white-opaque switching in *Candida albicans* are based on a self-sustaining transcriptional feedback loop. Proc Natl Acad Sci USA 103:12807–12812. doi:10.1073/pnas.0605138103 PMC1535343 16899543

[2] Huang G, Wang H, Chou S, Nie X, Chen J, Liu H. 2006. Bistable expression of *WOR1*, a master regulator of white–opaque switching in *Candida albicans*. Proc Natl Acad Sci USA 103:12813–12818. doi:10.1073/pnas.0605270103 PMC1540355 16905649

[3] Srikantha T, Borneman AR, Daniels KJ, Pujol C, Wu W, Seringhaus MR, Gerstein M, Yi S, Snyder M, Soll DR. 2006. TOS9 regulates white-opaque switching in *Candida albicans*. Eukaryot Cell 5:1674–1687. doi:10.1128/EC.00252-06 PMC1595353 16950924

[4] Nguyen VQ, Sil A. 2008. Temperature-induced switch to the pathogenic yeast form of *Histoplasma capsulatum* requires Ryp1, a conserved transcriptional regulator. Proc Natl Acad Sci USA 105:4880–4885. doi:10.1073/pnas.0710448105 PMC2290814 18339808

[5] Luo Z, Xiong D, Tian C. 2024. The roles of Gti1/Pac2 family proteins in fungal growth, morphogenesis, stress response, and pathogenicity. Mol Plant Microbe Interact 37:488–497. doi:10.1094/MPMI-11-23-0198-CR 38427716

[6] Lohse MB, Rosenberg OS, Cox JS, Stroud RM, Finer-Moore JS, Johnson AD. 2014. Structure of a new DNA-binding domain which regulates pathogenesis in a wide variety of fungi. Proc Natl Acad Sci USA 111:10404–10410. doi:10.1073/pnas.1410110111 PMC4115540 24994900

[7] Zhang S, Zhang T, Yan M, Ding J, Chen J. 2014. Crystal structure of the WOPR-DNA complex and implications for Wor1 function in white-opaque switching of *Candida albicans*. Cell Res 24:1108–1120. doi:10.1038/cr.2014.102 PMC4152732 25091450

[8] Román E, Prieto D, Hidalgo-Vico S, Alonso-Monge R, Pla J. 2023. The defective gut colonization of *Candida albicans hog1* MAPK mutants is restored by overexpressing the transcriptional regulator of the white opaque transition *WOR1*. Virulence 14:2174294. doi:10.1080/21505594.2023.2174294 PMC9928469 36760104

[9] Pande K, Chen C, Noble SM. 2013. Passage through the mammalian gut triggers a phenotypic switch that promotes *Candida albicans* commensalism. Nat Genet 45:1088–1091. doi:10.1038/ng.2710 PMC3758371 23892606

[10] Witchley JN, Basso P, Brimacombe CA, Abon NV, Noble SM. 2021. Recording of DNA-binding events reveals the importance of a repurposed *Candida albicans* regulatory network for gut commensalism. Cell Host Microbe 29:1002–1013. doi:10.1016/j.chom.2021.03.019 PMC8216204 33915113

[11] Liu OW, Chun CD, Chow ED, Chen C, Madhani HD, Noble SM. 2008. Systematic genetic analysis of virulence in the human fungal pathogen *Cryptococcus neoformans*. Cell 135:174–188. doi:10.1016/j.cell.2008.07.046 PMC2628477 18854164

[12] Cain CW, Lohse MB, Homann OR, Sil A, Johnson AD. 2012. A conserved transcriptional regulator governs fungal morphology in widely diverged species. Genetics 190:511–521. doi:10.1534/genetics.111.134080 PMC3276625 22095082

[13] Cromie GA, Tan Z, Hays M, Sirr A, Jeffery EW, Dudley AM. 2017. Transcriptional profiling of biofilm regulators identified by an overexpression screen in *Saccharomyces cerevisiae*. G3 (Bethesda) 7:2845–2854. doi:10.1534/g3.117.042440 PMC5555487 28673928

[14] Zordan RE, Miller MG, Galgoczy DJ, Tuch BB, Johnson AD. 2007. Interlocking transcriptional feedback loops control white-opaque switching in *Candida albicans*. PLoS Biol 5:e256. doi:10.1371/journal.pbio.0050256 PMC1976629 17880264

[15] Katsipoulaki M, Stappers MHT, Malavia-Jones D, Brunke S, Hube B, Gow NAR. 2024. *Candida albicans* and *Candida glabrata*: global priority pathogens. Microbiol Mol Biol Rev 88:e0002123. doi:10.1128/mmbr.00021-23 PMC11332356 38832801

[16] Rasheed M, Battu A, Kaur R. 2020. Host-pathogen interaction in *Candida glabrata* infection: current knowledge and implications for antifungal therapy. Expert Rev Anti Infect Ther 18:1093–1103. doi:10.1080/14787210.2020.1792773 32668993

[17] Healey KR, Jimenez Ortigosa C, Shor E, Perlin DS. 2016. Genetic drivers of multidrug resistance in *Candida glabrata*. Front Microbiol 7:1995. doi:10.3389/fmicb.2016.01995 PMC5156712 28018323

[18] Csank C, Haynes K. 2000. *Candida glabrata* displays pseudohyphal growth. FEMS Microbiol Lett 189:115–120. doi:10.1111/j.1574-6968.2000.tb09216.x 10913876

[19] Lachke SA, Joly S, Daniels K, Soll DR. 2002. Phenotypic switching and filamentation in *Candida glabrata*. Microbiology (Reading) 148:2661–2674. doi:10.1099/00221287-148-9-2661 12213913

[20] Kaur R, Domergue R, Zupancic ML, Cormack BP. 2005. A yeast by any other name: *Candida glabrata* and its interaction with the host. Curr Opin Microbiol 8:378–384. doi:10.1016/j.mib.2005.06.012 15996895

[21] Cormack BP, Ghori N, Falkow S. 1999. An adhesin of the yeast pathogen *Candida glabrata* mediating adherence to human epithelial cells. Science 285:578–582. doi:10.1126/science.285.5427.578 10417386

[22] Maestre-Reyna M, Diderrich R, Veelders MS, Eulenburg G, Kalugin V, Brückner S, Keller P, Rupp S, Mösch H-U, Essen L-O. 2012. Structural basis for promiscuity and specificity during *Candida glabrata* invasion of host epithelia. Proc Natl Acad Sci USA 109:16864–16869. doi:10.1073/pnas.1207653109 PMC3479469 23035251

[23] Zhang Y, Gong S, Xiong K, Yu X, Mo X, Su C, Lu Y. 2024. An alteration in the expression of cell wall structural proteins increases cell surface exposure of adhesins to promote virulence in *Candida glabrata*. mSphere 9:e0091024. doi:10.1128/msphere.00910-24 PMC11656777 39601558

[24] Srivastava VK, Suneetha KJ, Kaur R. 2014. A systematic analysis reveals an essential role for high-affinity iron uptake system, haemolysin and CFEM domain-containing protein in iron homoeostasis and virulence in *Candida glabrata*. Biochem J 463:103–114. doi:10.1042/BJ20140598 24987864

[25] Riedelberger M, Penninger P, Tscherner M, Seifert M, Jenull S, Brunnhofer C, Scheidl B, Tsymala I, Bourgeois C, Petryshyn A, Glaser W, Limbeck A, Strobl B, Weiss G, Kuchler K. 2020. Type I interferon response dysregulates host iron homeostasis and enhances *Candida glabrata* infection. Cell Host Microbe 27:454–466. doi:10.1016/j.chom.2020.01.023 32075740

[26] Gabaldón T, Carreté L. 2016. The birth of a deadly yeast: tracing the evolutionary emergence of virulence traits in *Candida glabrata*. FEMS Yeast Res 16:fov110. doi:10.1093/femsyr/fov110 PMC5815135 26684722

[27] Michielse CB, van Wijk R, Reijnen L, Manders EMM, Boas S, Olivain C, Alabouvette C, Rep M. 2009. The nuclear protein Sge1 of *Fusarium oxysporum* is required for parasitic growth. PLoS Pathog 5:e1000637. doi:10.1371/journal.ppat.1000637 PMC2762075 19851506

[28] Mo X, Yu X, Cui H, Xiong K, Yang S, Su C, Lu Y. 2024. *In vivo* RNA sequencing reveals a crucial role of Fus3-Kss1 MAPK pathway in *Candida glabrata* pathogenicity. mSphere 9:e0071524. doi:10.1128/msphere.00715-24 PMC11580445 39475321

[29] Luo G, Zhang J, Wang T, Cui H, Bai Y, Luo J, Zhang J, Zhang M, Di L, Yuan Y, Xiong K, Yu X, Zhang Y, Shen C, Zhu C, Wang Y, Su C, Lu Y. 2024. A human commensal-pathogenic fungus suppresses host immunity via targeting TBK1. Cell Host Microbe 32:1536–1551. doi:10.1016/j.chom.2024.07.003 39084229

[30] Timmermans B, De Las Peñas A, Castaño I, Van Dijck P. 2018. Adhesins in *Candida glabrata*. J Fungi (Basel) 4:60. doi:10.3390/jof4020060 PMC6023314 29783771

[31] Brunke S, Hube B. 2013. Two unlike cousins: *Candida albicans* and *C. glabrata* infection strategies. Cell Microbiol 15:701–708. doi:10.1111/cmi.12091 PMC3654559 23253282

[32] Ichikawa T, Kutsumi Y, Sadanaga J, Ishikawa M, Sugita D, Ikeda R. 2019. Adherence and cytotoxicity of *Candida* spp. to HaCaT and A549 cells. Med Mycol J 60:5–10. doi:10.3314/mmj.18-00011 30814468

[33] Denecker T, Zhou Li Y, Fairhead C, Budin K, Camadro JM, Bolotin-Fukuhara M, Angoulvant A, Lelandais G. 2020. Functional networks of co-expressed genes to explore iron homeostasis processes in the pathogenic yeast *Candida glabrata*. NAR Genom Bioinform 2:lqaa027. doi:10.1093/nargab/lqaa027 PMC7671338 33575583

[34] Nevitt T, Thiele DJ. 2011. Host iron withholding demands siderophore utilization for *Candida glabrata* to survive macrophage killing. PLoS Pathog 7:e1001322. doi:10.1371/journal.ppat.1001322 PMC3060170 21445236

[35] Vale-Silva L, Ischer F, Leibundgut-Landmann S, Sanglard D. 2013. Gain-of-function mutations in *PDR1*, a regulator of antifungal drug resistance in *Candida glabrata*, control adherence to host cells. Infect Immun 81:1709–1720. doi:10.1128/IAI.00074-13 PMC3648025 23460523

[36] Kaur R, Ma B, Cormack BP. 2007. A family of glycosylphosphatidylinositol-linked aspartyl proteases is required for virulence of *Candida glabrata*. Proc Natl Acad Sci USA 104:7628–7633. doi:10.1073/pnas.0611195104 PMC1863504 17456602

[37] Huang G, Yi S, Sahni N, Daniels KJ, Srikantha T, Soll DR. 2010. N-acetylglucosamine induces white to opaque switching, a mating prerequisite in *Candida albicans*. PLoS Pathog 6:e1000806. doi:10.1371/journal.ppat.1000806 PMC2837409 20300604

[38] Porman AM, Hirakawa MP, Jones SK, Wang N, Bennett RJ. 2013. MTL-independent phenotypic switching in *Candida tropicalis* and a dual role for Wor1 in regulating switching and filamentation. PLoS Genet 9:e1003369. doi:10.1371/journal.pgen.1003369 PMC3605238 23555286

[39] Phan-Canh T, Jenull S, Bitencourt T, Seiser S, Moser D, Motamedi M, Chowdhary A, Elbe-Bürger A, Kuchler K. 2025. White-Brown switching controls phenotypic plasticity and virulence of *Candida auris*. Cell Rep 44:115976. doi:10.1016/j.celrep.2025.115976 40638387

[40] Jonkers W, Dong Y, Broz K, Kistler HC. 2012. The Wor1-like protein Fgp1 regulates pathogenicity, toxin synthesis and reproduction in the phytopathogenic fungus *Fusarium graminearum*. PLoS Pathog 8:e1002724. doi:10.1371/journal.ppat.1002724 PMC3364952 22693448

[41] Qiu L, Zhang TS, Song JZ, Zhang J, Li Z, Wang JJ. 2021. BbWor1, a regulator of morphological transition, is involved in conidium-hypha switching, blastospore propagation, and virulence in *Beauveria bassiana*. Microbiol Spectr 9:e0020321. doi:10.1128/spectrum.00203-21 PMC8552717 34319134

[42] Lin X, Yang Y, Huang C, Xiong D, Qiu X, Tian C. 2025. Transcriptome analysis reveals that a Gti1/Pac2 family gene, *CpSge1*, regulates fungal growth, stress response, and virulence in *Cryphonectria parasitica*. Phytopathology 115:521–534. doi:10.1094/PHYTO-11-24-0354-R 39928834

[43] Vermitsky JP, Earhart KD, Smith WL, Homayouni R, Edlind TD, Rogers PD. 2006. Pdr1 regulates multidrug resistance in *Candida glabrata*: gene disruption and genome-wide expression studies. Mol Microbiol 61:704–722. doi:10.1111/j.1365-2958.2006.05235.x 16803598

[44] Tsai HF, Krol AA, Sarti KE, Bennett JE. 2006. *Candida glabrata PDR1*, a transcriptional regulator of a pleiotropic drug resistance network, mediates azole resistance in clinical isolates and petite mutants. Antimicrob Agents Chemother 50:1384–1392. doi:10.1128/AAC.50.4.1384-1392.2006 PMC1426987 16569856

[45] Torelli R, Posteraro B, Ferrari S, La Sorda M, Fadda G, Sanglard D, Sanguinetti M. 2008. The ATP-binding cassette transporter–encoding gene *CgSNQ2* is contributing to the *CgPDR1*-dependent azole resistance of *Candida glabrata*. Mol Microbiol 68:186–201. doi:10.1111/j.1365-2958.2008.06143.x 18312269

[46] Vale-Silva LA, Moeckli B, Torelli R, Posteraro B, Sanguinetti M, Sanglard D. 2016. Upregulation of the adhesin gene *EPA1* mediated by *PDR1* in *Candida glabrata* leads to enhanced host colonization. mSphere 1:e00065-15. doi:10.1128/mSphere.00065-15 PMC4863579 27303714

[47] Whaley SG, Caudle KE, Vermitsky JP, Chadwick SG, Toner G, Barker KS, Gygax SE, Rogers PD. 2014. *UPC2A* is required for high-level azole antifungal resistance in *Candida glabrata*. Antimicrob Agents Chemother 58:4543–4554. doi:10.1128/AAC.02217-13 PMC4136023 24867980

[48] Vu BG, Thomas GH, Moye-Rowley WS. 2019. Evidence that ergosterol biosynthesis modulates activity of the Pdr1 transcription factor in *Candida glabrata*. mBio 10:e00934-19. doi:10.1128/mBio.00934-19 PMC6561024 31186322

[49] Paul S, Bair TB, Moye-Rowley WS. 2014. Identification of genomic binding sites for *Candida glabrata* Pdr1 transcription factor in wild-type and ρ^0^ cells. Antimicrob Agents Chemother 58:6904–6912. doi:10.1128/AAC.03921-14 PMC4249425 25199772

[50] Vu BG, Stamnes MA, Li Y, Rogers PD, Moye-Rowley WS. 2021. The *Candida glabrata* Upc2A transcription factor is a global regulator of antifungal drug resistance pathways. PLoS Genet 17:e1009582. doi:10.1371/journal.pgen.1009582 PMC8509923 34591857

[51] Vyas VK, Bushkin GG, Bernstein DA, Getz MA, Sewastianik M, Barrasa MI, Bartel DP, Fink GR. 2018. New CRISPR mutagenesis strategies reveal variation in repair mechanisms among fungi. mSphere 3:e00154-18. doi:10.1128/mSphere.00154-18 PMC5917429 29695624

