# Supplementary material for: A WOPR transcriptional regulatory network controls the virulence of the opportunistic fungal pathogen Nakaseomyces glabratus
Source: Microbiol Spectr. 2026 Sep 22;14(11):e01865-26. doi: 10.1128/spectrum.01865-26 (PMC13729134; doi:10.1128/spectrum.01865-26)
Supplement: Supplementary file 1 [file spectrum.01865-26-s0001.pdf]

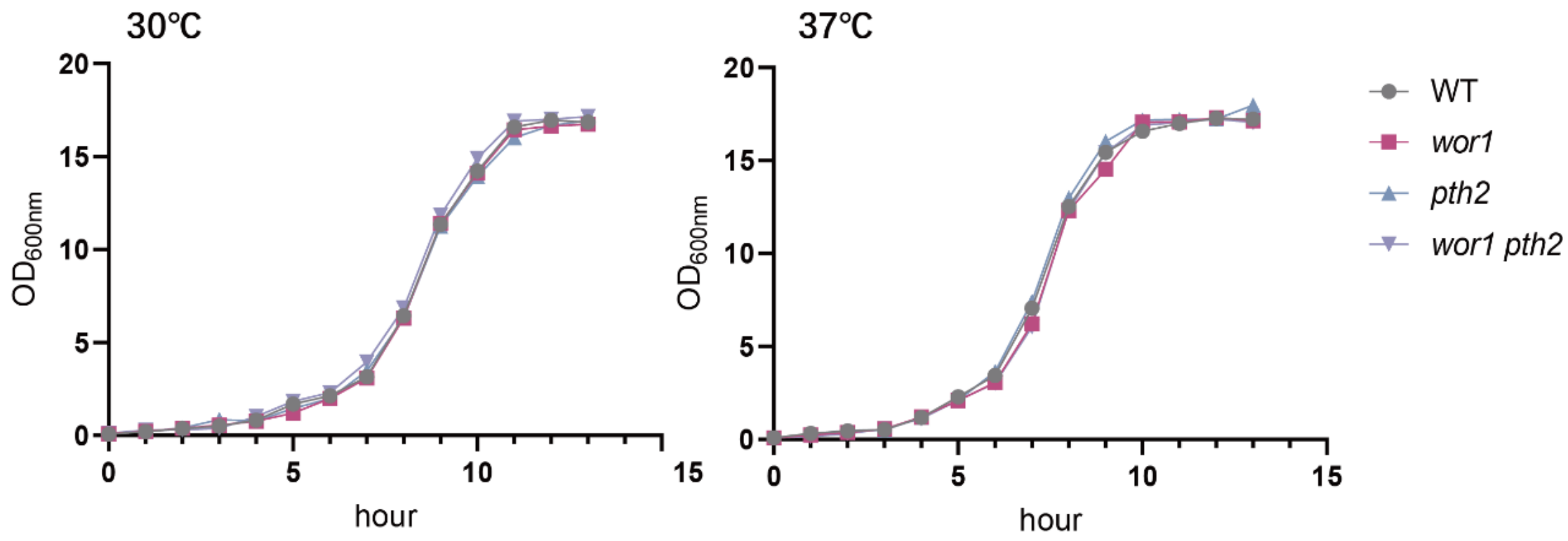

Figure S1. Growth assays for cells of wild type, single mutants *wor1* and *pth2*, and double mutant *wor1 pth2* incubated at 30°C or 37°C in liquid YPD medium. *N. glabratus* cells from overnight cultures were diluted into YPD medium to OD_{600} = 0.1 and then tested for growth.

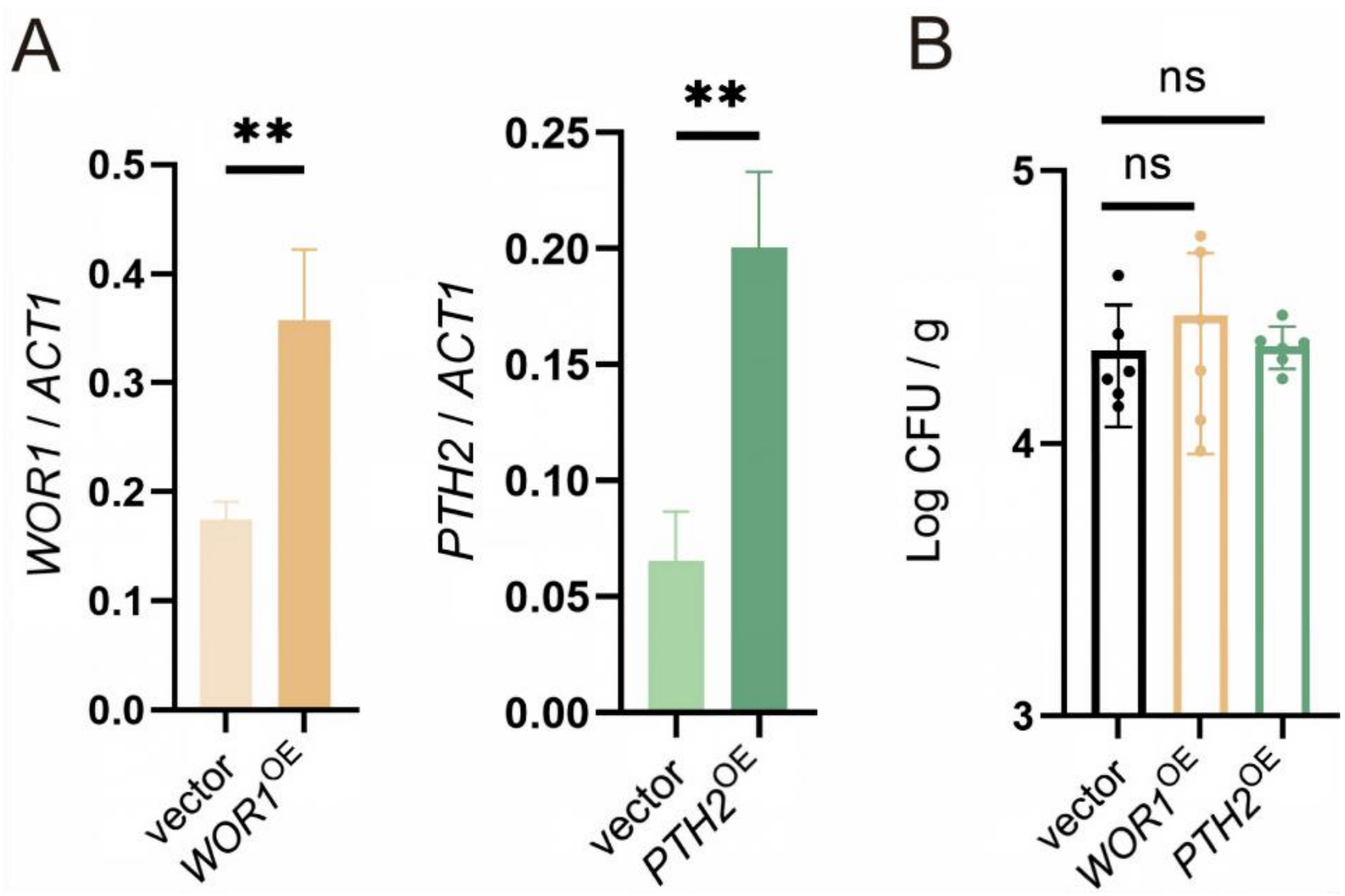

Figure S2. Impact of WOPR family gene overexpression on tissue colonization capacity of *N. glabratus in vivo*. A. Validation of gene overexpression by qRT-PCR in fungal cells recovered from mouse kidneys at 3 d post-infection. Relative transcript levels of targeted genes were measured in the *N. glabratus* strain CBS138 (WT) carrying pY26, pY26-WOR1, or pY26-PTH2. Expression levels are normalized to *ACT1*. Data represent mean ± SD from three independent replicates. B. Kidney fungal burden in systemically infected mice. Immunosuppressed BALB/c mice were intravenously infected via tail vein with 5×10^7 CFU of indicated strains. The experiment was performed as described in Figure 3A. Data represent means ± SD from six mice per group. Statistical significance was assessed by Student's t-test. ns, no significance; **, P < 0.01.

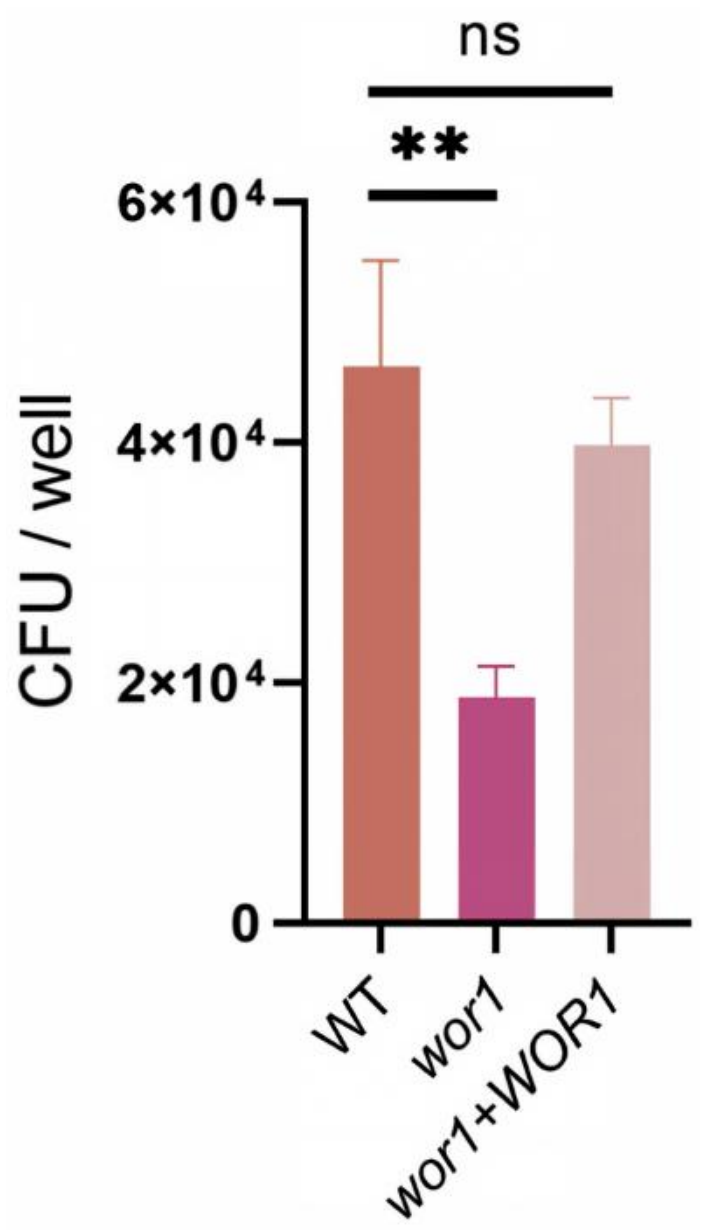

Figure S3. Adhesion assay of the *WOR1* complemented strain to HEK293 cells. Wild-type *N. glabratus, wor1* mutant strain or the *WOR1* complemented strain (*wor1*+pY26-WOR1) was co-cultured with HEK293 cells in DMEM medium supplemented with 10% fetal bovine serum for 4 h. Following incubation, non-adherent fungal cells were removed by washing with PBS. The number of adherent fungal cells was then quantified. Data are presented as means ± SD from three independent experiments.

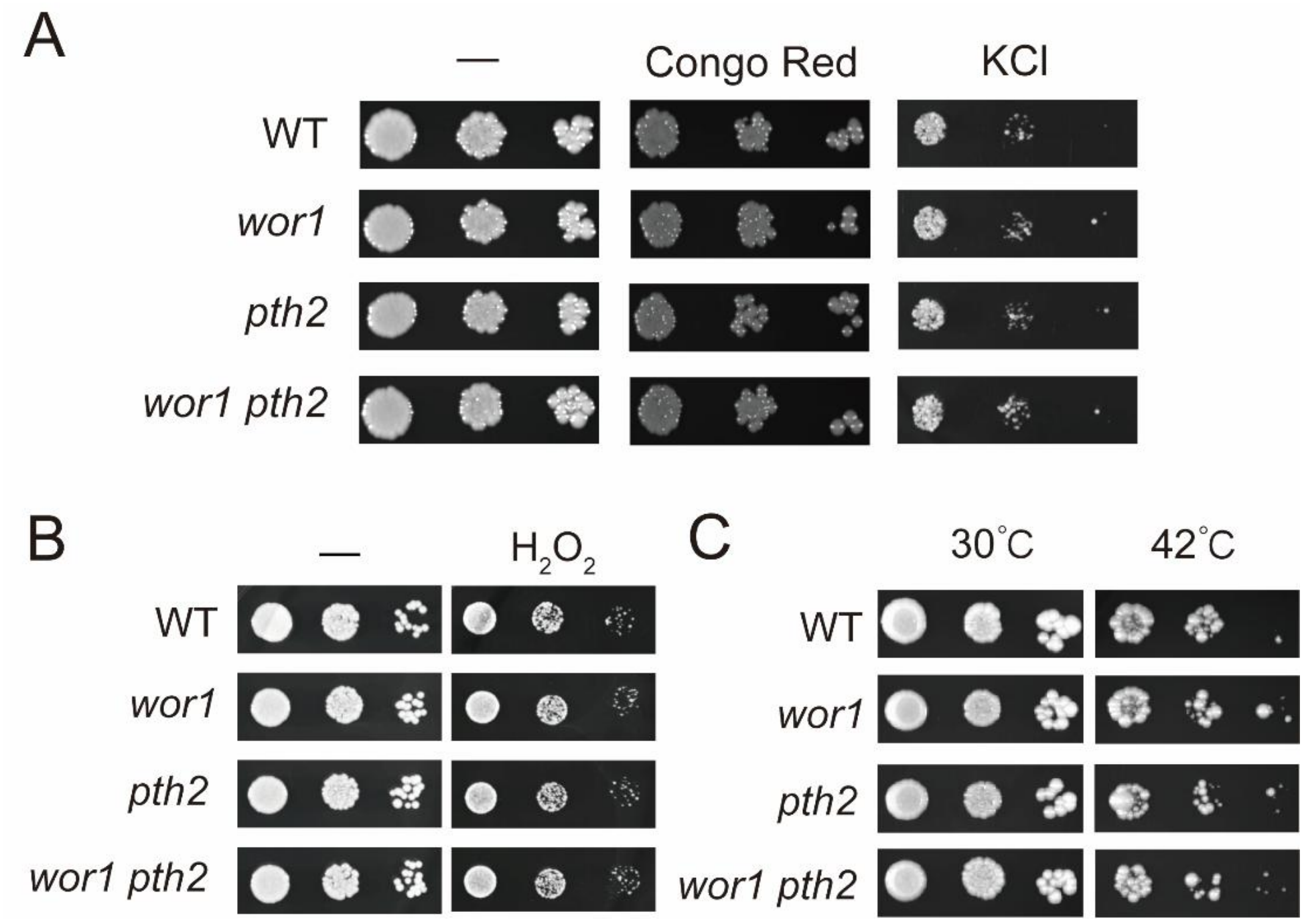

Figure S4. Effect of WOPR family gene deletion on *N. glabratus* stress response. A & B. WOPR genes are dispensable for stress resistance in *N. glabratus*. The indicated strains were exposed to various stressors: 4 mg/mL Congo red, 2.5 M KCl, and 10 mM H_2O_2. Photographs were taken after incubation at 30°C for 2 days to assess growth under these conditions. C. The WT strain and WOPR gene deletion mutants (*wor1*, *pth2*, and *wor1 pth2*) were subjected to spot dilution assays on YPD agar plates. Cultures were incubated at the standard growth temperature (30°C) and under the heat stress condition (42°C) for 4 days before photographs were taken.

A

Sample	Raw reads	Raw bases	Clean reads	Error rate(%)	Q30(%)	GC content(%)	Total map
WT-1	65811372	9937517172	65251198	0.0124	95.01	49.44	0.41%
WT-2	72909934	11009400034	72262316	0.0124	95.08	48.12	2.69%
wor1-1	74074912	11185311712	73422702	0.0118	96.31	48.9	0.03%
wor1-2	76835816	11602208216	76253808	0.0119	96.08	45.95	0.12%

B

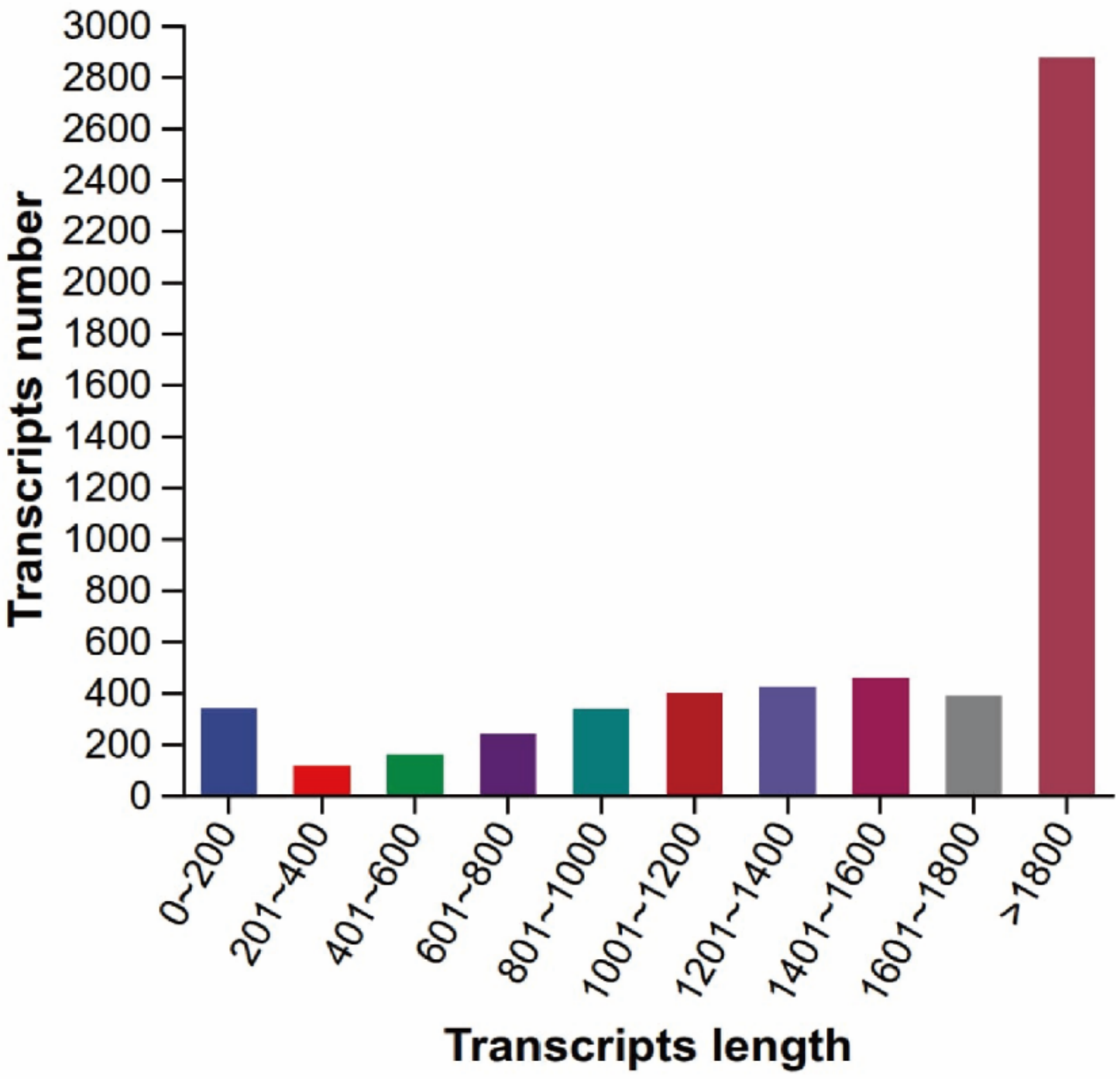

Figure S5. Quality assessment and length distribution of transcriptome sequencing data. A. Transcriptome sequencing data quality metrics. Summary statistics for each sample, including the number of raw reads, raw bases, number of clean (filtered) reads, sequencing error rate, proportion of Q30 bases, GC content distribution, and *N. glabratus* mapping rate are presented to evaluate the overall sequencing quality. Each group was represented by two biological replicates. B. Length distribution of fungal transcripts mapped to the *N. glabratus* genome. The x-axis represents transcript length intervals (in base pairs), and the y-axis denotes the absolute number of transcripts within each length category.

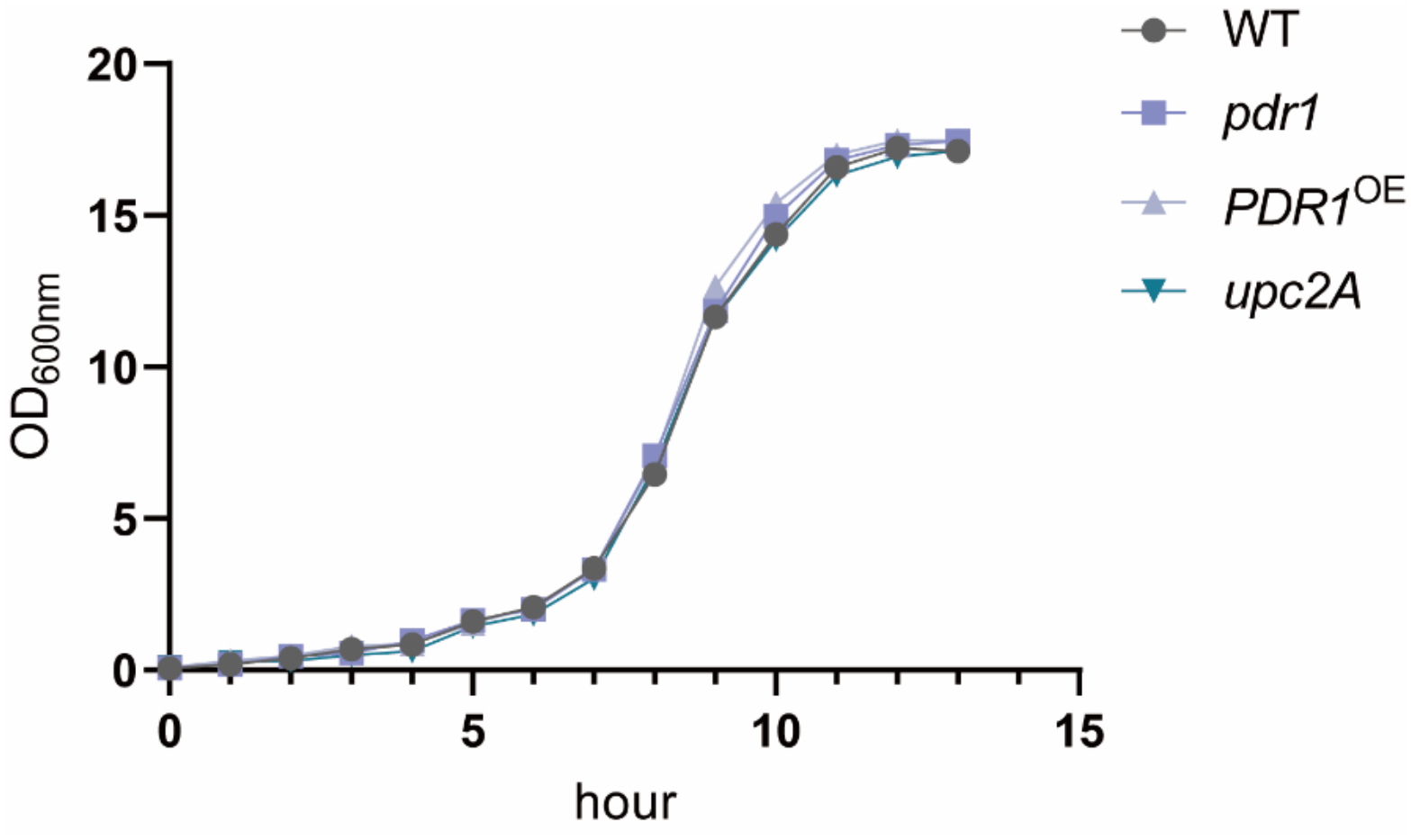

Figure S6. Growth analysis of WT and indicated strains in liquid medium at 30°C. Overnight cultures of WT, *PDR1* deletion, *PDR1* overexpression, and *UPC2A* deletion strains were diluted to an initial OD at 600 nm of 0.1 in YPD medium. Growth was monitored at 30°C to assess the growth of each strain.

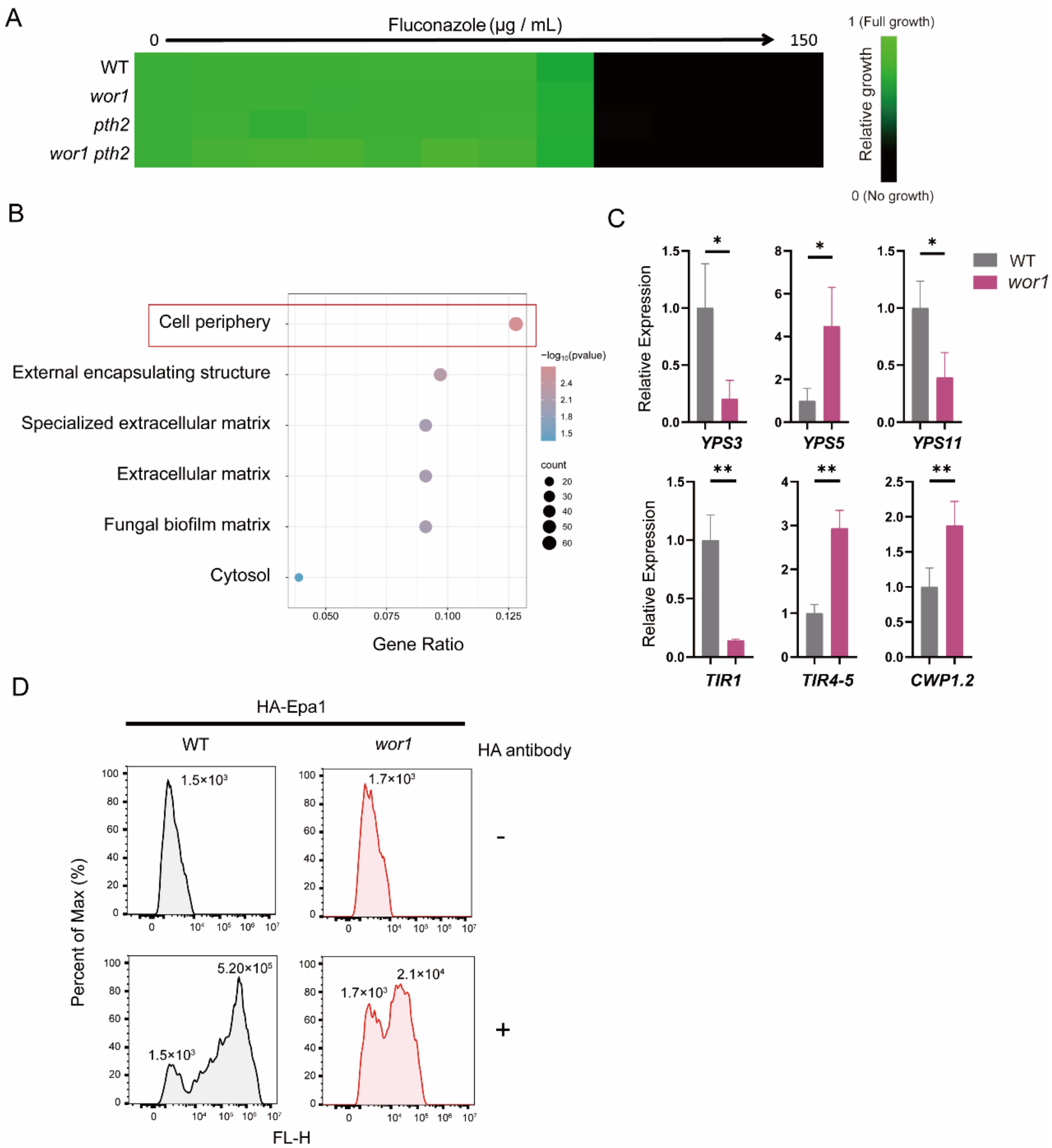

Figure S7. Enrichment of Wor1-regulated genes in the cell periphery. A. Fluconazole susceptibility assays were conducted in the YPD medium for wild-type and indicated mutant strains. Growth was measured by absorbance at 600 nm after 24 h at 30°C. Optical densities were normalized relative to those of antifungal drug-free controls. Data are quantitatively displayed in heat map format (see color bar). B. GO enrichment of differentially expressed genes between WT and *wor1* mutant within the Cellular Component (CC) category. The plot highlights key enriched GO terms related. The x-axis represents the Gene Ratio, and the y-axis lists the GO terms. Bubble size corresponds to the number of genes enriched in each category, while color reflects the significance of enrichment. C. qRT-PCR validation of Wor1-regulated genes associated with the cell periphery, including members of the YPS family and cell wall structural proteins, during invasive infection. Data are presented as means ± SD from three replicates. Statistical significance was assessed using an unpaired t-test with GraphPad Prism. *, $P < 0.05$; **, $P < 0.01$. D. Analysis of HA-

Epa1 fusion proteins on the cell surface in WT strain (black) and *wor1* mutant (red). *N. glabratus* cells carrying HA-Epa1 from the *TEF1* promoter were incubated in DMEM supplemented with 10% serum at 37°C in 5% CO_2. Surface exposure was monitored by immunofluorescence after labeling with anti-HA antibody and FITC-conjugated secondary goat anti-mouse antibody. No primary antibody control was included. Average fluorescence of the population was indicated. Plots are representative of data collected in three independent replicate experiments.

TABLE S2. ***N. glabratus*** **strains used in this study**

Strains	Genotype	Sources
CBS138	Wild type	ATCC collection
YLC166	*wor1Δ*	This study
YLC167	*pth2Δ*	This study
YLC168	*wor1Δ pth2Δ*	This study
YLC169	*pdr1Δ*	This study
YLC170	*PDR1pΔ*::*ScTEF1p*	This study
YLC171	*upc2AΔ*	This study

TABLE S3. **Primers used in this study**

	Sequence (5'-3')	Purpose and features
1	GATCGCAATGGAAAATACGACACTGG	Guide *WOR1*
2	AAAACCAGTGTCGTATTTTCCATTGC	
3	TTAATGAAGTAACTCCATCGGGTGAGTTGATTTAAGCAATGGAATTCACGTGACTGTAG	*WOR1* repair template
4	GAATGTCCACCGGAGGATCAAGTTTTAACGTTCCCCAGTCACGTGAATTCCATTGCTTA	
5	GATCGGGATGCACTTATCGTGCTTCG	Guide *PTH2*
6	AAAACGAAGCACGATAAGTGCATCCC	
7	ATTGCACCAACTTTTAAAGGCTACTTAGAGTCTGAGATCTAAGCACTTATCGTGCTTCA	*PTH2* repair template
8	TTCACAAACTTTAACTTCCCATCCAAGCAGCCCTGAAGCACGATAAGTGCTTAGATCTC	
9	GATCGAGGAGGAAAATAAAATGTAAG	Guide *PDR1*
10	AAAACTTACATTTTATTTTCCTCCTC	
11	AGAAGAACAAAAGTTGGAAAAGCTTGTGATAGCTGTAGAACGCGTTAAATAAAATGTAA	*PDR1* repair template
12	ACAGCCATAGATTGTACAAGATGGACAAGGTTTTAGCCCATTTACATTTTATTTTAAACGCG	
13	GATCGAAAACTCGGACGATAATACGG	Guide *UPC2A*
14	AAAACCGTATTATCGTCCGAGTTTTC	
15	TAAGTCCCCTGGTGCAAATAGCTTAGAAAACTCGGACGAGAATTCGTAACGATAAGTTT	*UPC2A* repair template
16	TTACTTTAGTATCGGGAGTAGATTGTGACAATTGTGGTGAAACTTATCGTTACGAATTC	
17	GATCGACACTAATTCCATATCACAAG	Guide *TEF1p*
18	AAAACTTGTGATATGGAATTAGTGTC	
19	TAACACATTCAAACTTCCATTACTTCGTACCCCATATCGTCATAGCTTCAAAATGTTTC	*TEF1p* repair template
20	CAATATATTCTAAAAAAAAAGGCAGAATAAAACACTAATTCTTAGATTAGATTGCTATG	
21	TAAGTTTTCTAGAACTAGCGCGGCCGCTTAAAGTGAAATGGACATAT	pY26-WOR1
22	GAATTGTTAATTAAAGATCTCCGCGGTTAAGATCTATTTTCGTTAG	
23	TAAGTTTTCTAGAACTAGCGCGGCCGCTAACAATTAATTCTTTCA	pY26-PTH2
24	GAATTGTTAATTAAAGATCTCCGCGGTTACGATGATTGAGATT	
25	GAAAGCATAGCAATCTAATCTAAGTTTTCTAGAATGGACATATCACCGACCTTTAAAGG	pYC121-WOR1
26	GCTTTTGTTCCACGTTAATTAACCGGATCCAAGATC	

	TATTTTCGTTAGGAACTTGCCCC	
27	AAATTCGCATAACAGTGCTG	*WOR1* qRT-PCR
28	AAAAGTGGACGTGGAGATGT	
29	TGTTTTCAGGGAAACACAGT	*PTH2* qRT-PCR
30	GCCTTCTCGTCAAATCTACCT	
31	CCAAAGTATGCAGCCTTGCC	*PDR1* qRT-PCR
32	GATCTGCAAGGTCAGGGCAT	
33	TTTCGAGTCCGGAAGTGTGG	*UPC2A* qRT-PCR
34	ATCCAGTCCCGGTTGTGTTC	
35	CTACGCACCAAAGCAAGTCG	*YPS3* qRT-PCR
36	CTGTTGTCCTTGGCGTTTGG	
37	GACTCCCAAGATCAGCGATG	*YPS5* qRT-PCR
38	CTTGCGCTGACAAACCACAA	
39	GGCTAAGAGGGCTGACGATG	*YPS11* qRT-PCR
40	GAGCACCAAGGACCCAGAAG	
41	TTGTTGCTGACTTCACCCCA	*CWP1.2* qRT-PCR
42	AGCTTGGATTTGACCGTCGT	
43	TTTCCGCTTTGGCTGCTTTC	*TIR1* qRT-PCR
44	GGTGTAGTCGGACAAGTGGG	
45	GCCACTGACGACTCCTACAC	*TIR4/TIR5* qRT-PCR
46	TTGGCCAACTTACCAGCCAA	
47	TCTAAGTTTTCTAGAACTAGCGCGGCCGCATGACAGCTGACATAGAGGATACTAAAGCC	pY26-UPC2A
48	CGAAGAATTGTTAATTAAAGATCTCCGCGGTTACATAAAATCCGACAAATTGGTCGTGG	
